# Neuropsychological outcomes comparing traditional surgical approaches and laser interstitial thermal therapy for refractory mesial temporal lobe epilepsy: A systematic review and meta‐analysis

**DOI:** 10.1111/epi.18687

**Published:** 2025-10-18

**Authors:** Konstantina Stavrogianni, Katerina Poprelka, Theodoros Fasilis, Vasileios Giannopapas, Wiebke Hahn, Iris Gorny, Aristotelis Kalyvas, Lampis Stavrinou, Sotirios Giannopoulos, Efstathios Boviatsis, Georgios Tsivgoulis, Anastasios Bonakis, Susanne Knake, Panagiota‐Eleni Tsalouchidou

**Affiliations:** ^1^ Second Department of Neurology Attikon University Hospital, National and Kapodistrian University of Athens Athens Greece; ^2^ Department of Physiology, Faculty of Medicine, School of Health Sciences University of Ioannina Ioannina Greece; ^3^ First Department of Neurosurgery National and Kapodistrian University of Athens Athens Greece; ^4^ Epilepsy Center Hessen, Department of Neurology Philipps University Marburg Marburg Germany; ^5^ Second Department of Neurosurgery Attikon University Hospital, School of Medicine, National and Kapodistrian University of Athens Athens Greece

**Keywords:** anterior temporal lobectomy, laser interstitial thermal therapy, mesial temporal lobe epilepsy, MRgLITT, naming, verbal memory, visual memory

## Abstract

**Objective:**

This systematic review and meta‐analysis aimed to compare neuropsychological outcomes following traditional surgical approaches and magnetic resonance‐guided laser interstitial thermal therapy (MRgLITT) in patients with drug‐resistant mesial temporal lobe epilepsy.

**Methods:**

Thirty‐four studies were included; 24 reported cognitive outcomes following open resection, seven following MRgLITT, and three reported on both procedures. Meta‐analyses of proportions, subgroup comparisons, and meta‐regressions were conducted across three cognitive domains: verbal memory, visual memory, and naming, stratified by surgical laterality and procedure type.

**Results:**

In left‐sided surgery, verbal memory decline occurred in 36% (95% confidence interval [CI] = 28%–45%) after open resection and 29% (95% CI = 9%–62%) after MRgLITT, with no significant difference (*p* = .5967). For right‐sided procedures, visual memory decline was similar between open resection (16%, 95% CI = 8%–29%) and MRgLITT (19%, 95% CI = 3%–61%; *p* = .8027). Left‐sided naming decline was higher after open resection (43%, 95% CI = 27%–61%) than MRgLITT (9%, 95% CI = 3%–22%; *p* < .0001), a difference supported in subgroup and meta‐regression analyses. Exploratory meta‐regression suggested a borderline association between higher seizure freedom and greater naming decline (*p* = .0508), but MRgLITT retained a protective effect after adjusting for seizure outcomes (*β* = −1.36, 95% CI = −2.20 to −.51; *p* = .0016). Regardless of the approach, naming improvement occurred in 27% of patients in each group after right‐sided surgery, indicating substantial recovery when the left hemisphere is preserved.

**Significance:**

Verbal memory showed a nonsignificant trend toward better preservation with MRgLITT, whereas visual memory was comparable across approaches. MRgLITT significantly preserved naming after left‐sided surgery, offering a clear cognitive benefit over traditional open resection.

**Key points**

- MRgLITT preserves naming better than open surgery in left‐sided mTLE.
- Verbal and visual memory outcomes were comparable across approaches.
- Higher seizure freedom rates trend toward more naming decline, suggesting a possible trade‐off.

## 1 INTRODUCTION

Epilepsy affects approximately 50 million people worldwide, making it one of the most common chronic neurological diseases.^1^ Approximately one third of patients develop drug‐resistant epilepsy, characterized by persistent seizures despite adequate trials of antiseizure medications.^2^ Temporal lobe epilepsy (TLE) is the most common focal epilepsy in adults, accounting for approximately 60% of all focal epilepsies.^3^ In patients with drug‐resistant TLE, surgical treatment offers significantly superior outcomes compared to continued medical therapy.^4^

Conventional surgical approaches, including anterior temporal lobectomy (ATL) and selective amygdalohippocampectomy (SAHE), have demonstrated seizure freedom rates of approximately 70%, along with improvements in quality of life and overall survival.^4^, ^5^, ^6^, ^7^ Over the past decade, magnetic resonance‐guided laser interstitial thermal therapy (MRgLITT) has emerged as a minimally invasive alternative to open resection, enabling real‐time, image‐guided ablation of the epileptogenic zone.^8^ This approach eliminates the need for open craniotomy and is associated with lower perioperative risk and faster postoperative recovery.^8^ Whereas early studies^9^ have shown seizure outcomes comparable to those reported in trials of ATL,^4^ recent meta‐analyses indicate that MRgLITT is associated with modestly lower seizure freedom rates than traditional open surgical approaches.^10^, ^11^

Although seizure control remains the primary objective of epilepsy surgery, neuropsychological outcomes are also important determinants of the postoperative quality of life of TLE patients.^12^, ^13^ Given the central role of the temporal lobe in memory, language, and affective cognitive networks,^14^ surgical resections inevitably carry a risk of cognitive compromise. These risks are strongly influenced by the laterality of the resection; procedures involving the left (dominant) temporal lobe are typically associated with declines in verbal memory and naming, whereas right (nondominant) resections more often impact visual memory.^15^, ^16^, ^17^

Neuropsychological outcomes following epilepsy surgery vary substantially depending on the type and extent of resection. Meta‐analytic and prospective studies demonstrate that conventional resections in the left (dominant) hemisphere are associated with up to ~40% risk of verbal memory and naming decline, whereas right (nondominant) resections more often result in visual memory deficits, typically at slightly lower rates.^15^, ^16^, ^17^, ^18^, ^19^, ^20^, ^21^ Selective approaches such as SAHE, which spare the lateral temporal neocortex, are associated with better preservation of cognitive function compared to broader resections like ATL that involve resection of functionally relevant neocortical areas.^22^ Recent studies suggest that neuropsychological outcomes following MRgLITT are generally favorable, showing lower rates of memory decline and greater preservation of language function compared to both ATL and SAHE.^23^, ^24^, ^25^, ^26^

Although previous meta‐analyses^10^, ^11^, ^27^ have included neuropsychological outcomes as secondary findings, their primary focus has remained on seizure control and surgical complications following MRgLITT, without directly comparing cognitive outcomes to those of conventional resective techniques. Therefore, this systematic review and meta‐analysis has as its primary outcome the evaluation of neuropsychological change, both decline and improvement, in verbal memory, visual memory, and naming. It directly compares traditional open resection and MRgLITT in mesial TLE (mTLE), using laterality‐specific data, standardized definitions of cognitive change, and meta‐regression to examine how cognitive outcomes relate to seizure control across surgical approaches.

## 2 MATERIALS AND METHODS

### 2.1 Search strategy

We systematically searched MEDLINE/PubMed, Embase, and Scopus for English‐language studies published up to June 15, 2025, using terms for anterior/temporal lobectomy, selective amygdalohippocampectomy, and laser interstitial thermal therapy (full strategies in Supplementary S1). Records were deduplicated in Mendeley. Two reviewers (K.S., K.P.) independently screened the titles, abstracts, and full texts; disagreements were resolved by a third reviewer (P.‐E.T.). Methods followed PRISMA (Preferred Reporting Items for Systematic Reviews and Meta‐Analyses) 2020 guidelines,^28^ and the protocol was registered in PROSPERO (International Prospective Register of Systematic Reviews; CRD420251114728).^29^

### 2.2 Eligibility criteria

Eligible studies were original, peer‐reviewed publications in English that included adults (≥16 years) with drug‐resistant mTLE who underwent open resection or MRgLITT. Studies were required to report the proportion of patients with decline or improvement in at least one domain (verbal memory, visual memory, or naming), stratified by surgical laterality, using validated methods for cognitive change including the Reliable Change Index, standardized regression‐based approaches, or study‐defined thresholds (e.g., change of ≥1 SD). For overlapping cohorts from the same institution, only the most comprehensive report was included. A minimum follow‐up of 6 months was required for postoperative outcomes.

### 2.3 Exclusion criteria

Studies were excluded if they were nonoriginal or non‐peer reviewed (e.g., case reports, reviews, editorials, conference abstracts), had a sample size < 5, involved pediatric‐only populations (<16 years old) without separately reported adult data, or presented overlapping cohorts without distinguishable data (based on institutional affiliation and recruitment periods). Also excluded were studies lacking laterality‐specific neuropsychological outcomes, those limited to nonresective or palliative procedures such as vagus nerve stimulation, corpus callosotomy, or responsive neurostimulation, and studies in which patients were treated with radiofrequency thermocoagulation.

### 2.4 Data extraction

Study‐ and patient‐level variables were extracted, including first author, year, sample size, sex distribution, age at epilepsy onset and surgery, surgical laterality, language dominance, and postoperative seizure outcomes (seizure freedom, Engel class I, or International League Against Epilepsy [ILAE] class 1). Neuropsychological outcomes were collected for verbal memory, visual memory, and naming, with proportions of patients showing decline or improvement stratified by laterality. When multiple tests assessed the same domain, one representative measure was selected, prioritizing delayed recall, larger sample size, prevalence in epilepsy research, and reviewer consensus. For studies with multiple follow‐up points, the 12‐month assessment or the time point linked to seizure outcomes was prioritized.

### 2.5 Risk of bias assessment

The risk of bias was evaluated with the ROBINS‐I tool, covering seven domains: confounding, participant selection, intervention classification, deviations from intended interventions, missing data, outcome measurement, and reporting bias. Two reviewers independently assessed each study (T.F. and V.G.), with disagreements resolved by a third reviewer (P.‐E.T.).

### 2.6 Meta‐analysis of proportions

Neuropsychological outcomes (verbal memory, visual memory, naming) were pooled using a random‐effects model with logit transformation. Results were stratified by intervention type, surgical laterality, and direction of change (decline or improvement). Because most studies reported outcomes by hemispheric laterality, terminology was standardized as “left” and “right” rather than “dominant” and “nondominant.” Studies reporting outcomes by language dominance were analyzed separately for the subgroup comparisons (see §2.7.1). Between‐study heterogeneity was assessed with τ^2^ and *I* ^2^, and 95% confidence intervals (CIs) were calculated using the Hartung–Knapp adjustment. All analyses were performed in R version 4.4.0 (2024‐04‐24) using the meta package (*metaprop* function).^30^, ^31^, ^32^ Forest plots present study‐level estimates with pooled proportions and CIs.

### 2.7 Comparison between traditional surgical approaches and MRgLITT for neuropsychological outcomes

#### 2.7.1 Subgroup analyses

Subgroup meta‐analyses compared open resection and MRgLITT for three domain–laterality pairings: verbal memory decline after left‐sided procedures, visual memory decline after right‐sided procedures, and naming decline after left‐sided procedures. These pairings reflect the functional organization of the temporal lobes, with the left hemisphere primarily supporting verbal memory and naming, and the right more commonly involved in visual memory processing. In addition, subgroup comparisons were conducted in studies only reporting outcomes by language dominance, excluding those limited to left/right laterality. Further subgroup comparisons for SAHE with MRgLITT were feasible only for verbal memory, as too few studies reported SAHE outcomes for other domains; subgroup comparisons for ATL with MRgLITT were conducted for verbal memory, visual memory, and naming. Finally, subgroup analyses based on stereoelectroencephalographic confirmation were not feasible, as these data were rarely reported. Analyses extended the primary meta‐analyses (Section 2.6) by stratifying data by surgical approach. Models were fitted with metaprop in the *meta* package, using a random‐effects model with logit transformation.^30^, ^31^, ^32^ Heterogeneity was quantified with τ^2^ and *I* ^2^, 95% CIs were calculated with the Hartung–Knapp adjustment, and subgroup differences were tested with the *χ* ^2^ test for interaction. Forest plots display study‐level estimates and pooled effects from the subgroup meta‐analyses, stratified by intervention type.

#### 2.7.2 Meta‐regression analyses

Meta‐regressions assessed whether surgical approach influenced outcomes for verbal memory decline (left‐sided), visual memory decline (right‐sided), and naming decline (left‐sided). In each model, surgical approach (MRgLITT vs. open resection) was included as a categorical moderator. The dependent variable was the logit‐transformed proportion of patients with decline, calculated using escalc in the *metafor* package. Mixed‐effects models were fitted using rma. Heterogeneity was quantified with τ^2^ and *I* ^2^, and moderator effects were summarized with *R* ^2^. Analyses were performed in R version 4.4.0.^30^, ^31^, ^32^

#### 2.7.3 Exploratory meta‐regression of naming decline and seizure freedom

An exploratory meta‐regression examined the relationship between naming decline and postoperative seizure freedom across approaches. Only studies reporting both outcomes after left‐sided procedures were included. The dependent variable was the logit‐transformed proportion of patients with naming decline. Seizure freedom (Engel class I) was modeled as a continuous moderator, and surgical approach (MRgLITT vs. open resection) as a categorical moderator. Two models were fitted with rma (*metafor* package): a main‐effects model (seizure freedom + approach) and an interaction model including an interaction term between seizure freedom and surgical approach. This interaction tested whether differences in naming decline between approaches were associated with seizure outcomes, reflecting a potential trade‐off between surgical efficacy and cognitive effects. Effect sizes were calculated with escalc. Analyses were performed in R version 4.4.0.^30^, ^31^, ^32^

## 3 RESULTS

### 3.1 Study selection and characteristics

**FIGURE 1:**
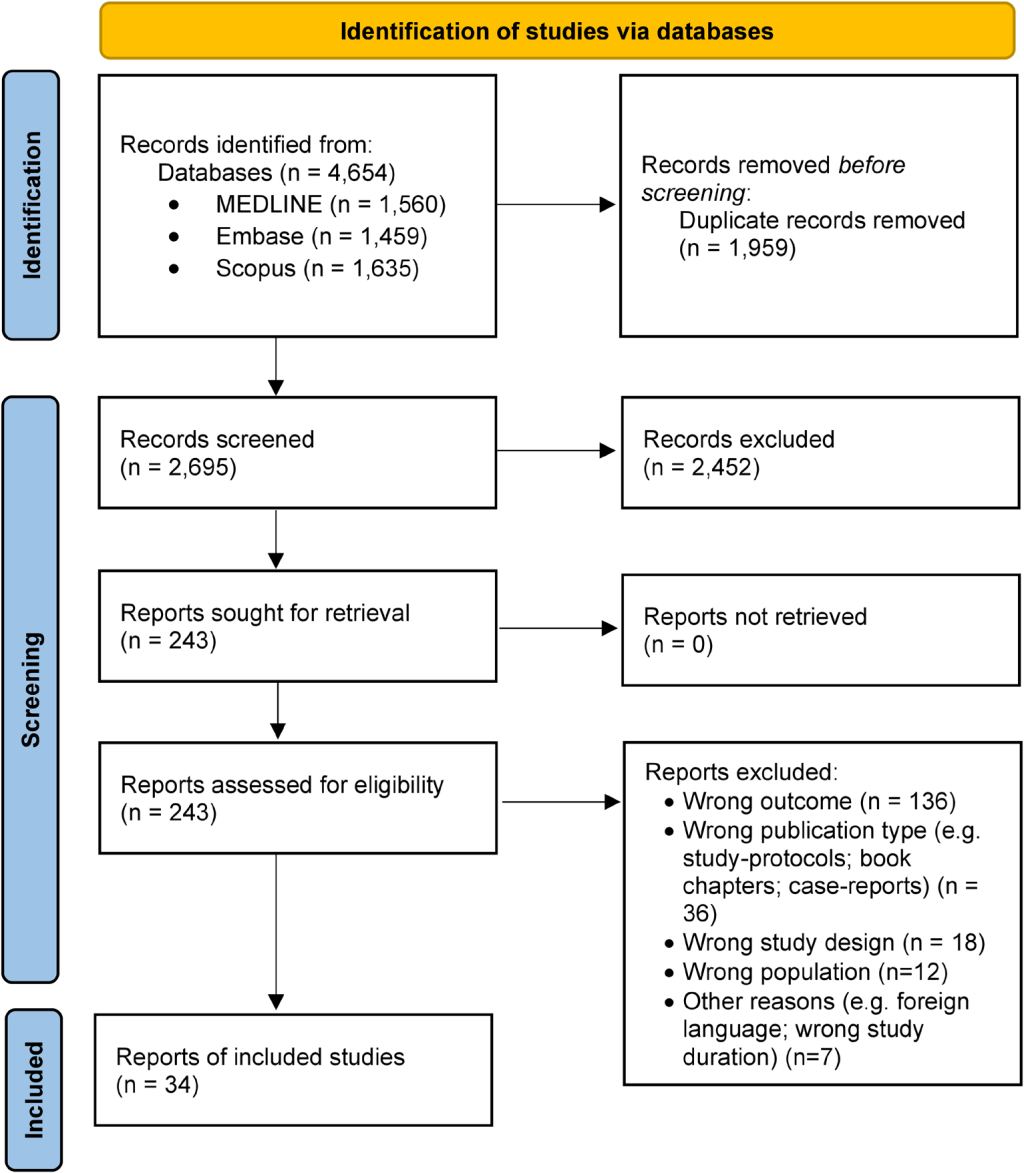
Flowchart of study selection process.

The database search (MEDLINE, Embase, Scopus) yielded 4654 records. After removing 1959 duplicates, 2695 titles/abstracts were screened, and 243 full texts were assessed. Thirty‐four studies met the inclusion criteria (Figure 1).

Among the included studies, 27 studies^19^, ^21^, ^33^, ^34^, ^35^, ^36^, ^37^, ^38^, ^39^, ^40^, ^41^, ^42^, ^43^, ^44^, ^45^, ^46^, ^47^, ^48^, ^49^, ^50^, ^51^, ^52^, ^53^, ^54^, ^55^, ^56^, ^57^ reported outcomes after traditional open resections (ATL, SAHE), and 10 studies^23^, ^24^, ^26^, ^54^, ^55^, ^56^, ^58^, ^59^, ^60^, ^61^ examined MRgLITT. Three contributed data to both groups. All studies reported surgical laterality, seizure outcomes, and neuropsychological change in at least one domain. Study characteristics (demographics, laterality, seizure freedom, design) are summarized in Table 1 and neuropsychological outcomes in Table 2.

**TABLE 1:** Overview of studies presenting neuropsychological outcomes with reliable change after traditional open resection procedures and MRgLITT.

Study	*N*	Mean/median age of epilepsy surgery, years (SD/range)	Mean/median age of epilepsy onset, years (SD/range)	Side of surgery, L/R, *n*	Postsurgical outcome, ILAE 1 or Engel I/IA		Study design
					Seizure‐free patients, ILAE 1, or Engel IA, *n* (%)	Engel I, *n* (%)	
Traditional open resection							
Chelune et al., 1993	96	L: 29.4 (7.6) R: 29.4 (7.3)	L: 12.5 (9.2) R: 13.8 (9.2)	L = 47; R = 49	NA	L: 37 (78.8%) R: 39 (83.7%)	Prospective
Davies et al., 1998	99	31.4 (9.4)	11.8 (10.3)	L = 99; R = 0	NA	NA	Retrospective
Martin et al., 1998	101	L: 32.2 (9.3) R: 30.0 (8.8)	L: 11.9 (9.1) R: 12.3 (9.2)	L = 53; R = 48	NA	70 (69%)	Retrospective
Sawrie et al., 1999	65	34.6 (11.2)	–	L = 20; R = 25	NA	NA	Prospective
Helmstaedter et al., 2003	147 SAHE: 33%	31 (10)	12 (9)	L = 72; R = 75	93 (63%)	NA	Prospective
Stroup et al., 2003	132	37.3 (9.8)	–	L = 58; R = 74	NA	78 (54%)	Retrospective
Engman et al., 2004	54	Median: 34 (25.3–43.8)	Median: 16.5 (6.3–25.0)	L = 25; R = 29	L: 16 (64%); R: 19 (66%)	NA	Prospective
Baxendale & Thompson, 2005	290	L: 31.1 (8.3) R: 32.2 (9.0)	L: 10.8 (9.2) R: 11.7 (9.1)	L = 157; R = 133	NA	190 (65.5%)	Retrospective
Engman et al., 2006	25	Median: 30 (26.0–42.0)	Median: 14 (4.0–22.0)	L = 10; R = 15	NA	14 (58%)	Retrospective
Lineweaver et al., 2006	87	33.5 (12.4)	–	L = 37; R = 50	NA	66 (77%)	Retrospective
Paglioli et al., 2006	161 SAHE: 81	31.3 (8–62)	7.3 (1–35)	L = 93; R = 68	116 (72%)	NA	Prospective
Dulay et al., 2009	75	L: 29.5 (7.8) R: 31.5 (10.4)	L: 13.2 (8.7) R: 14.4 (8.9)	L = 33; R = 42	NA	60 (80%)	Prospective
Grammaldo et al., 2009	82	34.1 (10.3)	–	L = 35; R = 47	63 (77%)	NA	Prospective
Drane et al., 2015	39 SAHE: 17	D: 36.0 (11.4) ND: 36.5 (11.4)	D: 16.7 (12.9) ND: 13.9 (9.3)	L = 22; R = 17	NA	24 (61.5%)	Prospective
Giovagnoli et al., 2016	106	L: 36.9 (11.1) R: 34.3 (10.6)	L: 17.3 (12.4) R: 17.1 (13.0)	L = 54; R = 52	L: 44 (81%) R: 45 (87%)	NA	Prospective
Nascimento et al., 2016	67 SAHE: 34	35.5 (−)	9.2 (−)	L = 34; R = 33	ATL: 18 (54.5%) SAHE: 23 (64.7%)	NA	Retrospective
Cano‐López et al., 2017	61	40.3 (12.7)	14.3 (10.8)	L = 34; R = 27	NA	53 (86.9%)	Prospective
Pauli et al., 2017	48	33.3 (10.7)	11.6 (6.9)	L = 21; R = 27	33 (68.8%)	NA	Prospective
Barbaro et al., 2018	27	41.3 (12.9)	9.96 (10.9)	L = 12; R = 15	NA	NA	Prospective
Drane et al., 2021	40 SAHE: 13	D: 36.9 (12.4) ND: 39.2 (2.2)	D: 21.2 (14.2) ND: 18.8 (12.3)	L = 19; R = 21	NA	18 (45%)	Prospective
Hebel et al., 2021	51 SAHE: 37%	Median: 57.3 (50–73.6)	Median: 15 (<1–62)	L = 22; R = 24	30 (43%)	NA	Retrospective
Sone et al., 2022	142	L: 36.7 (11.8) R: 37.4 (11.2)	L: 12.2 (9.6) R: 14.1 (10.5)	L = 74; R = 68	L: 40 (54%) R: 37 (54%)	NA	Prospective
Castro‐Lima et al., 2023	43	L: 34.4 (9.5) R: 34.5 (8.9)	L: 8.1 (4.6) R: 14.8 (8.5)	L = 9; R = 10	L: 8 (88.9%) R: 7 (70%)	NA	Prospective
Hageboutros et al., 2024	22 SAHE: 11	ATL: 34.4 (10.0) SAHE: 37.6 (14.2)	ATL: 16.7 (10.8) SAHE: 20.2 (18.4)	L = 22	14 (63.6%)	NA	Retrospective
Fleury et al., 2024	25	L: 38 (13) R: 38 (21)	NA	L = 12; R = 13	L: 10 (83%) R: 12 (92%)	NA	Retrospective
Mezjan et al., 2025	34	32.9 (11)	13.2 (2 months–38 years)	L = 34	NA	25 (73.5%)	Retrospective
Sablik et al., 2025	24	L: 37.5 (13.6) R: 39.5 (17.5)	L: 19 (12.8) R: 15.5 (10.8)	L = 12; R = 12	20 (83%)	NA	Retrospective
MRgLITT							
Drane et al., 2015	19	D: 38.2 (17.1) ND: 36.2 (13.3)	D: 12.4 (12.1) ND: 15.4 (1.9)	L = 10; R = 9	NA	11 (57.9%)	Prospective
Kang et al., 2016	20 NPS: 6	51.2 (−)	24.8 (−)	L = 5; R = 1	4 (36.4%)	NA	Prospective
Tao et al., 2018	21 NPS: 10	40 (13)	22 (−)	L = 10; R = 11 NPS: L = 7; R = 3	11 (52%)	NA	Prospective
Jermakowicz et al., 2017	23 NPS: 20	40.9 (−)	12 (−)	L = 12; R = 11 NPS: L = 10; R = 10	8 (34.8%)	NA	Retrospective
Greenway et al., 2017	15	44.6 (−)	NA	L = 9; R = 6	5 (33.3%)	NA	Retrospective
Gross et al., 2018	58 NPS: 49	40.4 (15.1)	17.1 (14.2)	L = 28; R = 30 NPS: L = 20; R = 29	22 (37.9%)	NA	Retrospective
Bermudez et al., 2020	26	D: 43.7 (14.5) ND: 44.9 (11.7)	D: 13.9 (15.8) ND: 17.0 (11.0)	L = 14; R = 12 NPS: L = 11; R = 9	NA	21 (81%)	Retrospective
Drane et al., 2021	40	D: 39.7 (17.0) ND: 40.7 (14.3)	D: 14.6 (10.1) ND: 18.7 (14.9)	L = 19; R = 21	NA	13 (32.5%)	Prospective
Hageboutros et al., 2024	11	43.5 (16.5)	15.5 (10.3)	L = 11	NA	7 (64%)	Retrospective
Infante et al., 2025	17	39.6 (−)	11.6 (−)	L = 15; R = 2	NA	11 (64%)	Retrospective

Abbreviations: ATL, anterior temporal lobectomy; D, dominant hemisphere; ILAE, International League Against Epilepsy; L, left hemisphere; MRgLITT, magnetic resonance‐guided laser interstitial thermal therapy; NA, not available; ND, nondominant hemisphere; NPS, neuropsychological data available; R, right hemisphere; SAHE, selective amygdalohippocampectomy.

**TABLE 2:** Neuropsychological outcomes and reliable change metrics following traditional open resection and MRgLITT.

Study	Left		Right		Performed test	Statistical method/criterion used to define cognitive change	Follow‐up, months
	Improved, *n*	Declined, *n*	Improved, *n*	Declined, *n*			
Verbal memory							
Traditional open resection							
Chelune et al., 1993	0	21	1	7	WMS‐R Verbal Memory subtests	RCI	10.8
Martin et al., 1998	8	26	15	16	WMS Logical Memory subtest, CVLT	SRB	12
Sawrie et al., 1999	2	9	3	7	CVLT	SRB	6
Stroup et al., 2003	0	27	0	8	CVLT, WMS‐R Logical Memory subtest	RCI	12
Helmstaedter et al., 2003	9	32	8	18	German version of the VLMT	RCI	58
Engman et al., 2004	1	9	5	7	CM Paired Word Test	RCI	24
Baxendale & Thompson, 2005	9	34	13	15	AMIPB	RCI	12
Engman et al., 2006	1	4	3	1	CM Memory Test	RCI	30
Lineweaver et al., 2006	0	14	NA	9	WMS‐III	>1 SD	10.2
Paglioli et al., 2006	9	9	7	2	WMS‐R, RAVLT	>1 SD	12
Dulay et al., 2009	9	21	18	12	VSRT	RCI	11.8
Grammaldo et al., 2009	1	2	2	0	RAVLT, Story Recall	RCI	24
Nascimento et al., 2016	12	23	NA	NA	RAVLT	*z*‐score; >1 SD	12
Cano‐López et al., 2017	6	15	8	3	Spanish version of the CVLT (TAVEC)	RCI	12
Barbaro et al., 2018	NA	3	NA	NA	CVLT, WMS Logical Memory subtest	RCI	36
Drane et al., 2021	2	9	1	2	RAVLT	RCI	6
Hebel et al., 2021	1	4	4	3	German version of the RAVLT	Δz ≥ | ± 1|^a^	≈34.8 (2.9 years)
Sone et al., 2022	NA	24	NA	12	BIRT List Learning	RCI	12
Castro‐Lima et al., 2023	NA	4	NA	NA	RAVLT	RCI	12
Fleury et al., 2024	6	4	4	3	BMIPB Verbal Learning subtests	RCI	120
MRgLITT							
Kang et al., 2016	0	3	0	0	CVLT‐II, WMS‐IV Logical Memory I & II subtests	RCI	≈11.5 (348 days)
Tao et al., 2018	0	2	0	0	CVLT‐II, WMS Logical Memory subtest	RCI	8.4
Jermakowicz et al., 2017	1	2	3	1	WMS Logical Memory subtest	>1 SD	6
Greenway et al., 2017	0	9	0	5	CVLT, WMS‐IV Logical Memory subtest, WMS‐IV Verbal Paired Associates subtest	RCI	6
Gross et al., 2018	2	3	7	1	RAVLT	RCI	13
Bermudez et al., 2020	3	1	3	0	WMS Logical Memory subtest, RAVLT, Miami Assessment of Memory Instrument	>1 SD	6.4
Drane et al., 2021	1	2	3	0	RAVLT	RCI	12
Infante et al., 2025	0	4	0	0	FCSRT	RCI	10.2
Visual memory							
Traditional open resection							
Chelune et al., 1993	3	4	3	5	WMS‐R Visual Memory subtests	RCI	10.8
Martin et al., 1998	13	12	7	12	WMS Visual Reproduction subtest	SRB	12
Helmstaedter et al., 2003	9	20	9	26	DCS‐R	RCI	58
Engman et al., 2004	4	6	3	5	ROCFT, TCFT	RCI	24
Engman et al., 2006	1	4	1	2	ROCFT	RCI	30
Lineweaver et al., 2006	NA	10	NA	14	WMS‐III	>1 SD	10.2
Dulay et al., 2009	6	4	5	17	NVSRT	RCI	11.8
Grammaldo et al., 2009	1	0	2	0	ROCFT	RCI	24
Nascimento et al., 2016	12	2	NA	NA	ROCFT	*z*‐score, >1 SD	12
Hebel et al., 2021	5	1	5	2	DCS‐II	Δz ≥ | ± 1|^a^	≈34.8 (2.9 years)
Sone et al., 2022	NA	16	NA	7	BIRT Design Learning subtest	RCI	12
Fleury et al., 2024	5	6	5	5	BMIPB Design Learning subtests	RCI	120
MRgLITT							
Jermakowicz et al., 2017	2	2	3	1	BVMT‐R	>1 SD	6
Greenway et al., 2017	0	4	0	3	WMS‐IV Visual Reproduction subtest	RCI	6
Bermudez et al., 2020	1	1	1	1	BVMT‐R, RCFT	>1 SD	6.4
Infante et al., 2025	0	0	0	0	ROCFT	RCI	10.2
Naming							
Traditional open resection							
Davies et al., 1998	NA	31	NA	NA	BNT, VN subtest	RCI	6
Martin et al., 1998	2	21	2	0	BNT	SRB	12
Drane et al., 2015	NA	21	NA	11	BNT, IFF	RCI	6
Giovagnoli et al., 2016	9	15	23	0	BNT	RCI	24
Pauli et al., 2017	NA	7	NA	1	BNT	1 SD	12–14
Cano‐López et al., 2017	6	13	9	2	BNT	RCI	12
Hageboutros et al., 2024	1	13	NA	NA	BNT	RCI	≈11.2 (341 days)
Mezjan et al., 2025	8	11	NA	NA	DO‐80	RCI	18
Sabik et al., 2025	6	4	6	4	GNT	RCI	108
MRgLITT							
Drane et al., 2015	NA	0	NA	0	BNT, IFF	RCI for BNT; >1 SD for IFF	8
Tao et al., 2018	0	2	0	0	BNT	RCI	8.4
Jermakowicz et al., 2017	2	2	3	1	BNT	>1 SD	6
Greenway et al., 2017	1	0	1	1	BNT	RCI	6
Bermudez et al., 2020	2	1	4	1	BNT	>1 SD	6.4
Hageboutros et al., 2024	4	0	NA	NA	BNT	RCI	12
Infante et al., 2025	0	1	0	0	BNT	RCI	10.2

Abbreviations: AMIPB, Adult Memory and Information Processing Battery; BIRT, Brain Injury Rehabilitation Trust battery; BMIPB, BIRT Memory and Information Processing Battery, a revision and extension of the AMIPB; BNT, Boston Naming Test; BVMT‐R, Brief Visual Memory Test‐Revised; CM, Crohnholm–Molander Memory Test; CVLT, California Verbal Learning Test; CVLT‐II, California Verbal Learning Test, Second Edition; DCS‐II, Diagnostikum für Zerebralschadigung, Version II; DCS‐R, Diagnostikum für Zerebralschadigung, Revised; DO‐80, Test de dénomination orale d'images 80; FCSRT, Free and Cued Selective Reminding Test; GNT, McKenna Graded Naming Test; IFF, Iowa Famous Faces Test; MRgLITT, magnetic resonance‐guided laser interstitial thermal therapy; NA, not available; NVSRT, Non‐Verbal Selective Reminding Test; RAVLT, Rey Auditory Verbal Learning Test; RCFT, Rey Complex Figure Test; RCI, Reliable Change Index; ROCFT, Rey–Osterrieth Complex Figure Test; SRB, standardized regression‐based change scores; TAVEC, Test de Aprendizaje Verbal España‐Complutense, a Spanish version of the CVLT; TCFT, Taylor Complex Figure Test; VLMT, Verbal Learning and Memory Test (German version: Verbaler Lern‐ und Merkfähigkeitstest); VN, Visual Naming; VSRT, Verbal Selective Reminding Test; WMS, Wechsler Memory Scale; WMS‐III, Wechsler Memory Scale, Third Edition; WMS‐IV, Wechsler Memory Scale, Fourth Edition; WMS‐R, Wechsler Memory Scale, Revised.

^a^ Δz represents the change in z‐scores between post‐ and preoperative assessments.

### 3.2 Risk of bias assessment

The overall risk of bias across included studies was predominantly low to moderate. Traditional resection studies showed low risk in intervention classification, outcome measurement, and reporting (Figure S2.1). MRgLITT studies demonstrated a similar pattern, with generally low risk and only moderate concerns for confounding or missing data (Figure S2.2). Full assessments for all studies are presented in Supplementary Material S2.

### 3.3 Neuropsychological outcomes following traditional surgical approaches

#### 3.3.1 Verbal memory

Following left‐sided open surgery, the pooled rate of verbal memory decline was 36% (95% CI = 28%–45%; 21 studies, *n* = 856; *I* ^2^ = 77.8%; Figure S3.1.1), whereas improvement was less frequent at 8% (95% CI = 4%–15%; 17 studies, *n* = 761; *I* ^2^ = 62.3%; Figure S3.1.2). Right‐sided resections showed a pooled decline rate of 14% (95% CI = 10%–20%; 19 studies, *n* = 808; *I* ^2^ = 51.1%) and a pooled improvement rate of 12% (95% CI = 7%–21%; 16 studies, *n* = 690; *I* ^2^ = 71.4%; Figures S3.1.3–S3.1.4).

#### 3.3.2 Visual memory

Following left‐sided open surgery, the pooled rate of visual memory decline was 16% (95% CI = 10%–27%; 11 studies, *n* = 454; *I* ^2^ = 50.3%; Figure S3.2.1). The pooled rate of visual memory improvement was similar at 17% (95% CI = 10%–26%; 10 studies, *n* = 343; *I* ^2^ = 57.7%; Figure S3.2.2). For right‐sided resections, the pooled rate of visual memory decline was also 16% (95% CI = 8%–29%; 10 studies, *n* = 410; *I* ^2^ = 67.2%; Figure S3.2.3), whereas the pooled improvement rate was 12% (95% CI = 8%–17%; nine studies, *n* = 342; *I* ^2^ = 39.5%; Figure S3.2.4).

#### 3.3.3 Naming

Following left‐sided open surgery, the pooled rate of naming decline was 43% (95% CI = 27%–61%; nine studies, *n* = 351; *I* ^2^ = 60.8%; Figure S3.3.1). Naming improvement was observed in 15% (95% CI = 6%–34%; six studies, *n* = 209; *I* ^2^ = 67.9%; Figure S3.3.2).

For right‐sided surgery, the pooled rate of naming decline was 5% (95% CI = 0–52%; six studies, *n* = 183; *I* ^2^ = 73.7%; Figure S3.3.3), whereas improvement was reported in 27% (95% CI = 5%–71%; four studies, *n* = 139; *I* ^2^ = 80.2%; Figure S3.3.4).

### 3.4 Neuropsychological outcomes following MRgLITT

#### 3.4.1 Verbal memory

Following left‐sided MRgLITT, the pooled rate of verbal memory decline was 29% (95% CI = 9%–62%; eight studies, *n* = 96; *I* ^2^ = 0%; Figure S4.1.1). Verbal memory improvement was observed in 8% (95% CI = 3%–19%; eight studies, *n* = 83; *I* ^2^ = 0%; Figure S4.1.2). For right‐sided MRgLITT, the pooled rate of verbal memory decline was 3% (95% CI = 0%–49%; eight studies, *n* = 81; *I* ^2^ = 40.3%; Figure S4.1.3). Improvement was more frequent, observed in 20% (95% CI = 11%–32%; eight studies, *n* = 81; *I* ^2^ = 0%; Figure S4.1.4).

#### 3.4.2 Visual memory

Following left‐sided MRgLITT, the pooled proportion of visual memory decline was 15% (95% CI = 2%–62%; four studies, *n* = 41; *I* ^2^ = 0%; Figure S4.2.1). Improvement in visual memory was less frequent, observed in 7% (95% CI = 1%–48%; four studies, *n* = 41; *I* ^2^ = 0%; Figure S4.2.2). For right‐sided MRgLITT, the pooled proportion of visual memory decline was 19% (95% CI = 3%–61%; four studies, *n* = 26; *I* ^2^ = 14.8%; Figure S4.2.3). The pooled improvement rate was 12% (95% CI = 2%–48%; four studies, *n* = 26; *I* ^2^ = 0%; Figure S4.2.4).

#### 3.4.3 Naming

Following left‐sided MRgLITT, the pooled proportion of naming decline was 9% (95% CI = 3%–22%; seven studies, *n* = 68; *I* ^2^ = 0%; Figure S4.3.1). Naming improvement was reported in 14% (95% CI = 4%–38%; six studies, *n* = 58; *I* ^2^ = 0%; Figure S4.3.2). For right‐sided MRgLITT, the pooled proportion of naming decline was 8% (95% CI = 2%–28%; six studies, *n* = 39; *I* ^2^ = 0%; Figure S4.3.3), whereas improvement was observed in 27% (95% CI = 10%–53%; five studies, *n* = 30; *I* ^2^ = 0%; Figure S4.3.4).

### 3.5 Comparison between traditional surgical procedures and MRgLITT for neuropsychological outcomes

#### 3.5.1 Comparison of verbal memory decline for left‐sided procedures

**FIGURE 2:**
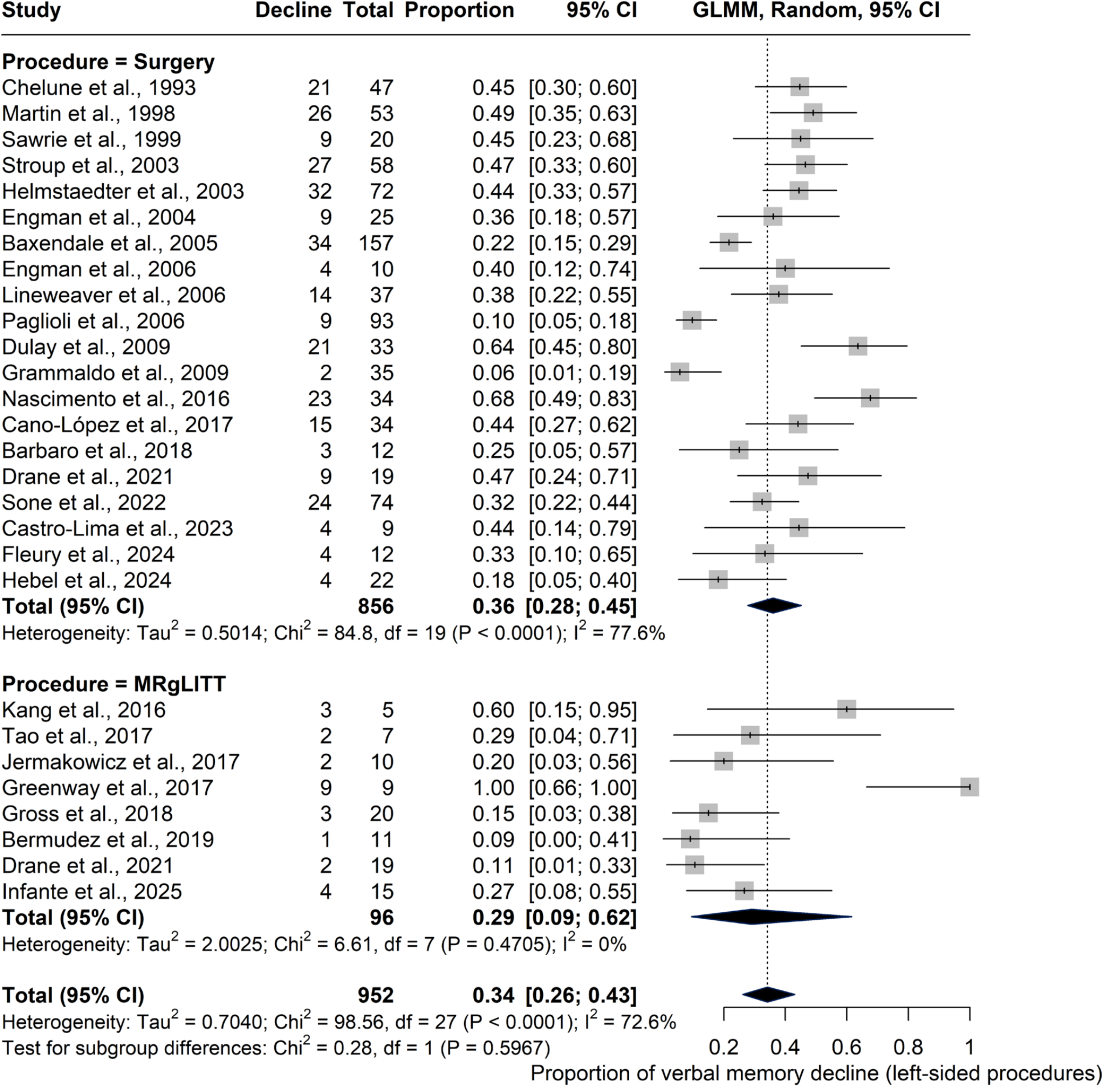
Forest plot showing the proportion of verbal memory decline following left‐sided procedures. Squares represent individual study estimates with 95% confidence intervals (CIs), scaled by study weight. Diamonds indicate pooled proportions for traditional open surgery and magnetic resonance‐guided laser interstitial thermal therapy (MRgLITT), as well as the overall estimate. Subgroup analysis by procedure type was performed using a random‐effects model. The pooled decline was .36 for open surgery and .29 for MRgLITT. The test for subgroup differences was not statistically significant (*χ* ^2^ = .28, *p* = .5967). GLMM, generalized linear mixed model.

Subgroup analysis comparing left‐sided procedures suggested a trend toward lower rates of verbal memory decline with MRgLITT compared to traditional open surgery, although the difference was not statistically significant (*χ* ^2^ = .28, *df* = 1, *p* = .597). Meta‐regression confirmed this pattern; the intercept, representing MRgLITT, was significant (*β* = −.54, SE = .17, *p* = .0016, 95% CI = −.87 to −.20), indicating a consistent baseline proportion of decline in this group. The coefficient for surgery versus MRgLITT was not significant (*β* = −.54, SE = .41, *p* = .182, 95% CI = −1.34 to .25), suggesting no robust difference between the two approaches. The subgroup meta‐analysis is shown in Figure 2, and full meta‐regression results are available in Table S5.1.

#### 3.5.2 Comparison of visual memory decline for right‐sided procedures

The subgroup analysis of right‐sided interventions included 14 studies (*n* = 436). Open surgery (10 studies, *n* = 410) yielded a pooled decline rate of 16% (95% CI = 8%–29%; *I* ^2^ = 67.2%), whereas MRgLITT (four studies, *n* = 26) showed a pooled decline of 19% (95% CI = 3%–61%; *I* ^2^ = 14.8%). The difference between groups was not statistically significant (*χ* ^2^ = .06, *df* = 1, *p* = .803).

**FIGURE 3:**
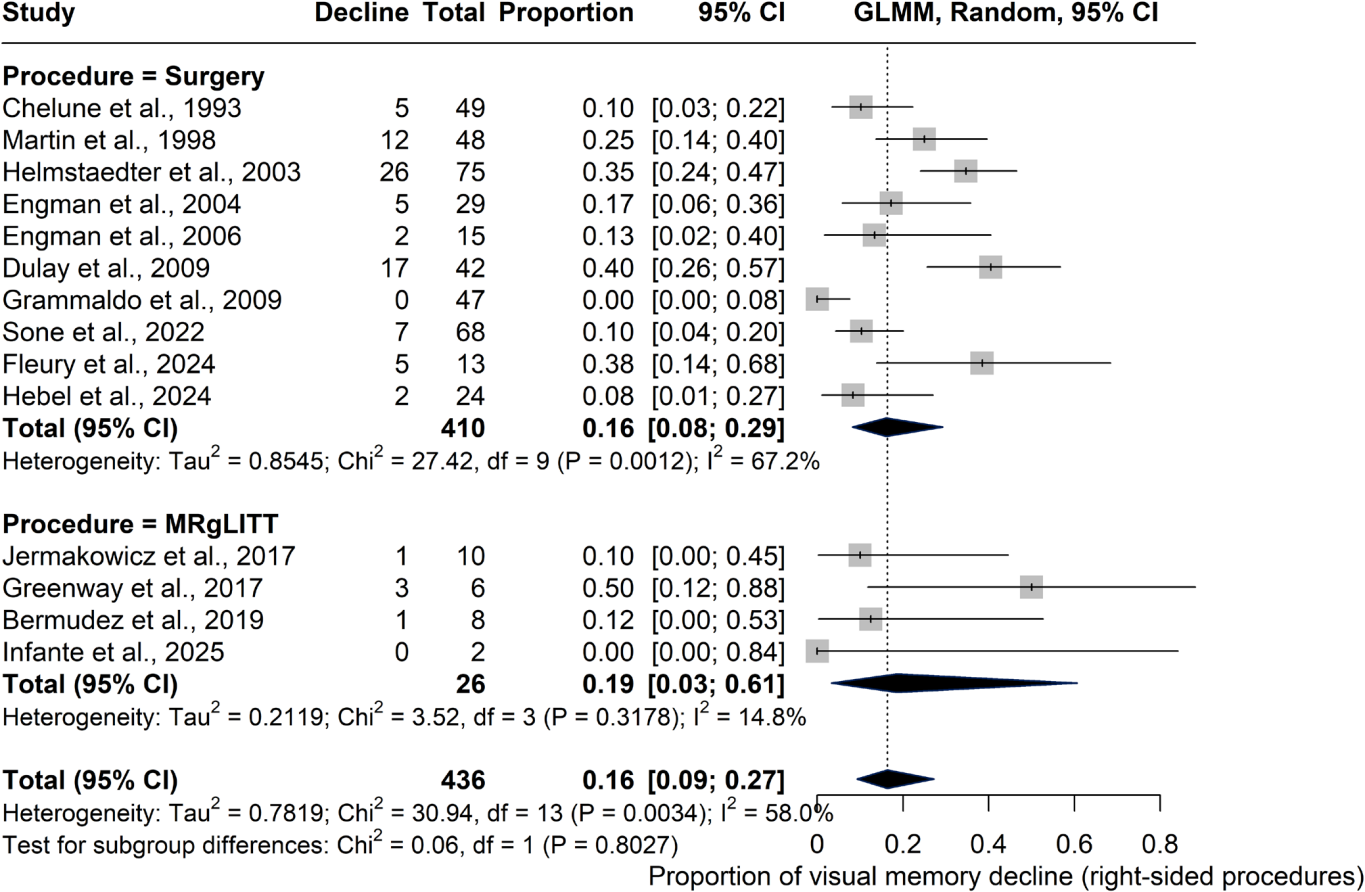
Forest plot displaying the proportion of visual memory decline following right‐sided procedures. Each square represents an individual study, along with its corresponding 95% confidence interval (CI). Diamonds indicate pooled proportions for the open surgery and magnetic resonance‐guided laser interstitial thermal therapy (MRgLITT) subgroups, as well as the overall estimate. Subgroup analysis was performed using a random‐effects model. The pooled decline was .16 for open surgery and .19 for MRgLITT. The test for subgroup differences was not statistically significant (*χ* ^2^ = .06, *p* = .8027). GLMM, generalized linear mixed model.

Meta‐regression results were consistent; the intercept for MRgLITT was significant (*β* = −1.26, SE = .62, *p* = .041, 95% CI = −2.48 to −.05), indicating a measurable baseline rate of decline in this group, whereas the coefficient for open surgery versus MRgLITT was not (*β* = −.15, SE = .67, *p* = .828, 95% CI = −1.46 to 1.17), supporting no significant difference between approaches. The subgroup meta‐analysis is shown in Figure 3, and full meta‐regression results are provided in Table S5.2.

#### 3.5.3 Comparison of naming for left‐sided procedures

**FIGURE 4:**
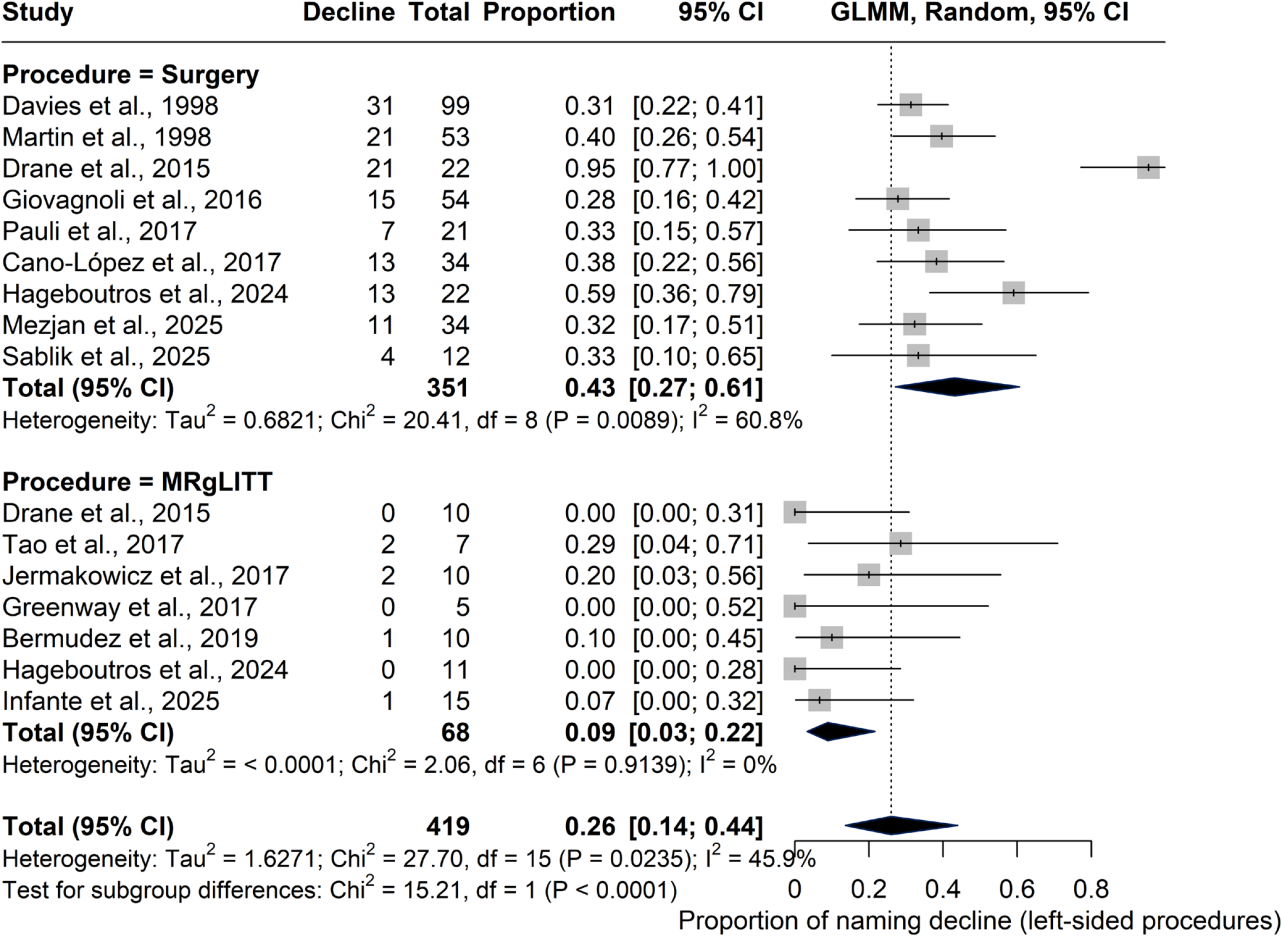
Forest plot showing the proportion of naming decline after left‐sided procedures. Each square represents an individual study and its 95% confidence interval (CI), scaled by study weight. Diamonds indicate pooled subgroup estimates for open surgery and magnetic resonance‐guided laser interstitial thermal therapy (MRgLITT), as well as the overall estimate. The pooled decline was .43 (95% CI = .27–.61) for traditional surgery and .09 (95% CI = .03–.22) for MRgLITT. The difference between subgroups was statistically significant (*χ* ^2^ = 15.21, *p* < .0001). GLMM, generalized linear mixed model.

The subgroup analysis included 16 studies (*n* = 419), with 351 patients undergoing open surgery and 68 MRgLITT. The pooled decline rate was 43% (95% CI = 27%–61%; *I* ^2^ = 60.8%) for open surgery and 9% (95% CI = 3%–22%; *I* ^2^ = 0%) for MRgLITT. The difference was statistically significant (*χ* ^2^ = 15.21, *df* = 1, *p* < .0001), indicating a lower proportion of naming decline with MRgLITT (Figure 4).

Meta‐regression confirmed procedure type as a significant moderator. The MRgLITT intercept showed a consistently low baseline decline (*β* = −1.92, SE = .40, *p* < .0001, 95% CI = −2.71 to −1.13), whereas open surgery was associated with higher rates (*β* = 1.37, SE = .42, *p* = .0012, 95% CI = .54–2.19) and only low residual heterogeneity (*I* ^2^ = 6.2%). These results indicate that MRgLITT is associated with better preservation of naming function in left‐sided procedures compared with open surgery. Full meta‐regression outputs are available in Table S5.3.

#### 3.5.4 Association between naming decline and seizure freedom across surgical approaches

A total of 14 studies reporting both naming decline and seizure freedom rates after left‐sided procedures were included in the exploratory meta‐regression. In the main model, higher seizure freedom rates showed a positive association with greater naming decline, reaching borderline statistical significance (*β* = .015, 95% CI = .00–.029, *p* = .0508). After adjusting for seizure outcomes, MRgLITT remained independently associated with lower rates of naming decline compared with open surgery (*β* = −1.36, 95% CI = −2.20 to −.51, *p* = .0016). The model explained nearly all between‐study variation (*R* ^2^ = 98.9%), with minimal residual heterogeneity (*I* ^2^ = 2.8%).

In the secondary interaction model, the association between seizure freedom and naming decline did not differ significantly by surgical approach (interaction: *β* = −1.63, *p* = .59). This suggests that the reduced naming decline observed with MRgLITT is unlikely to be explained by differences in seizure control. Both models showed good fit with low unexplained variability. Full results are presented in Tables S5.4 and S5.5.

#### 3.5.5 Subgroup comparisons by language dominance

In studies explicitly reporting outcomes by language dominance, subgroup analyses comparing open resection and MRgLITT were conducted for dominant verbal memory, nondominant visual memory, and dominant naming. For dominant verbal memory, the pooled decline rate was 43% (95% CI = 36%–51%; *I* ^2^ = 47.8%) after open resection and 27% (95% CI = 2%–85%; *I* ^2^ = 11.2%) after MRgLITT, with no significant difference between approaches (*χ* ^2^ = .49, *p* = .48). For nondominant visual memory, the pooled decline rate was 18% (95% CI = 11%–29%; *I* ^2^ = 67.1%) after open resection and 12% (95% CI = 1%–64%; *I* ^2^ = 14.8%) after MRgLITT, with no significant difference between approaches (*χ* ^2^ = .34, *p* = .56). For dominant naming, the pooled decline rate was 48% (95% CI = 26%–70%; *I* ^2^ = 67.3%) after open resection and 8% (95% CI = 2%–25%; *I* ^2^ = 0%) after MRgLITT, showing a statistically significant advantage for MRgLITT (*χ* ^2^ = 13.19, *p* = .0003). These findings were consistent with the laterality‐based analyses. The corresponding forest plots are presented in Figures S6.1–S6.3.

#### 3.5.6 Subgroup comparison of SAHE and MRgLITT

For left‐sided procedures, two studies of SAHE (*n* = 72) and eight studies of MRgLITT (*n* = 96) reported proportions of patients with verbal memory decline. The pooled decline rate was 22% (95% CI = 0–100%; *I* ^2^ = 88.8%) after SAHE and 27% (95% CI = 10%–51%; *I* ^2^ = 42.5%) after MRgLITT. The difference between procedures was not statistically significant (*χ* ^2^ = .19, *p* = .67). The corresponding forest plot is presented in Figure S6.4.

#### 3.5.7 Subgroup comparison of ATL and MRgLITT

In the subgroup comparison between ATL and MRgLITT, verbal memory decline after left‐sided procedures was reported in 16 ATL studies (*n* = 639) and eight MRgLITT studies (*n* = 96), with pooled rates of 37% (95% CI = 26%–50%; *I* ^2^ = 76.1%) and 29% (95% CI = 9%–62%; *I* ^2^ = 0%), respectively, showing no significant difference (*χ* ^2^ = .33, *p* = .57). For visual memory decline after right‐sided procedures, 10 ATL studies (*n* = 285) and four MRgLITT studies (*n* = 26) reported pooled rates of 16% (95% CI = 8%–28%; *I* ^2^ = 61.1%) and 19% (95% CI = 3%–61%; *I* ^2^ = 14.8%), again without a significant difference (*χ* ^2^ = .09, *p* = .76). Similar to the previous analyses, naming decline after left‐sided procedures was reported in seven ATL studies (*n* = 295) and seven MRgLITT studies (*n* = 68), with pooled rates of 35% (95% CI = 28%–42%; *I* ^2^ = 21.3%) and 9% (95% CI = 3%–22%; *I* ^2^ = 0%), respectively, showing a significant difference in favor of MRgLITT (*χ* ^2^ = 14.57, *p* = .0001). The corresponding forest plots are shown in Figures S6.5–S6.7.

## 4 DISCUSSION

The present meta‐analysis compared neuropsychological outcomes after open resection and MRgLITT in drug‐resistant mTLE, stratified by laterality. For verbal memory, left‐sided procedures were associated with a substantially higher rate of impairment, with pooled estimates of 36% in patients following open resection and 29% after MRgLITT. This difference was not statistically significant in the subgroup and meta‐regression analyses, indicating a trend toward better outcomes with MRgLITT but no clear superiority over traditional approaches for verbal memory, a finding that was unchanged when analyses were restricted to studies explicitly reporting dominant‐hemisphere procedures. These findings are consistent with previous literature. Sherman et al.^15^ reported a pooled rate of 44% after left ATL, whereas Alomar et al.^27^ found a pooled rate of 31.8% after MRgLITT. Minor discrepancies in pooled estimates likely reflect differences in the statistical models. Similarly, MRgLITT showed no advantage for verbal memory improvement in left‐sided interventions, with improvement rates of 8% after both open resection and MRgLITT. For right‐sided procedures, verbal memory losses occurred in 14% after open resection and 3% after MRgLITT, whereas improvement was observed in 12% and 20%, respectively. These rates align with previous literature showing relatively preserved verbal memory following right‐sided procedures, with declines typically below 20%.^15^, ^21^, ^27^

MRgLITT showed no advantage over open resection for visual memory decline after right‐sided procedures in both subgroup and meta‐regression analyses, with rates of 16% for open resection and 19% for MRgLITT. Although right‐sided, nondominant hemisphere procedures are traditionally considered more likely to cause visual memory impairment, previous meta‐analyses^15^, ^27^ and our findings showed no clear lateralization in this domain. In open resection, pooled decline rates were identical for both hemispheres (16%), and in MRgLITT rates were similarly close (19% for right‐sided vs. 15% for left‐sided procedures). Overall, considering the limited number of MRgLITT studies reporting visual memory outcomes, rates were generally low across lobes, with no substantial differences observed between surgical approaches or hemispheres.

Visual memory gains were uncommon across both hemispheres; after right‐sided procedures, improvement occurred in 12% of patients following either open resection or MRgLITT, whereas for left‐sided procedures, the rates were 17% and 7%, respectively. Although the removal of a dysfunctional temporal lobe may enable functional compensation,^21^ these findings suggest that the smaller ablation volumes of MRgLITT do not provide a measurable advantage for visuospatial memory recovery.

For naming, left‐sided procedures revealed a marked difference between approaches. The pooled decline rate after open resection was 43% compared to 9% after MRgLITT, a statistically significant difference in the subgroup analysis (*p* < .0001). Meta‐regression confirmed these findings, indicating that MRgLITT is associated with a consistently low rate of naming deterioration. These results were similar in the subgroup of studies limited to dominant‐hemisphere procedures, suggesting that, unlike verbal and visual memory, naming ability after dominant‐hemisphere procedures is significantly better preserved with MRgLITT.

Recent meta‐analyses have reported that MRgLITT achieves slightly lower seizure freedom rates than conventional surgical approaches.^10^, ^11^ Procedures involving lower tissue volume, such as MRgLITT, may spare unaffected functional areas, leading to better preservation of naming performance,^22^ yet they may also carry a higher likelihood of leaving parts of the epileptogenic zone untreated, potentially compromising seizure outcomes. Invasive evaluations such as stereoelectroencephalography can more precisely localize the epileptogenic zone and help evaluate seizure outcomes, particularly for MRgLITT, but such data were not sufficiently reported in the included studies to allow analysis. In this meta‐analysis, a borderline association (*p* = .0508) was observed between higher seizure freedom rates and greater naming impairment, consistent with a potential compromise between seizure control and naming preservation. Nevertheless, MRgLITT remained associated with lower naming decline even after adjusting for seizure outcomes, suggesting that factors beyond tissue volume, such as surgical trajectory or reduced collateral injury, may also contribute to its cognitive advantage.

Interestingly, naming improvement reached 27% in both open resection and MRgLITT after right‐sided procedures, suggesting that, irrespective of the approach, sparing the dominant (left) hemisphere allows for substantial postoperative language recovery. Verbal memory also showed a notable pooled improvement rate after right‐sided procedures. These findings contrast with the generally lower improvement rates observed for verbal and visual memory overall, indicating that language functions may benefit more from compensatory reorganization when the dominant hemisphere remains intact.^62^ Such recovery is likely supported by preserved left‐hemisphere language networks and adaptive neuroplasticity, combined with the cessation of epileptic activity, which may also have a secondary positive impact on naming performance.^21^

## 5 LIMITATIONS

This meta‐analysis has some limitations. First, the number of studies reporting neuropsychological outcomes after MRgLITT remains limited, particularly for right‐sided procedures, resulting in smaller pooled sample sizes for the examined cognitive domains. Second, follow‐up durations varied among studies, which may have influenced both seizure and cognitive outcomes. Third, although only studies applying rigorous, objectively defined criteria for cognitive change were included, neuropsychological outcomes may still vary according to the specific tests and scoring methods used. Fourth, the data were insufficient to allow a comparison of selective approaches (SAHE vs. MRgLITT) in the significant domain of naming, as the proportions of patients with naming decline after SAHE were not consistently reported. Fifth, seizure outcome definitions varied across studies; Engel class I and ILAE class 1 were grouped for the analysis as reported in the original studies, which may have introduced variability. Finally, the degree of between‐study heterogeneity differed across cognitive domains, likely reflecting differences in study design, patient characteristics, and methods of outcome assessment.

## 6 CONCLUSIONS

In this systematic review and meta‐analysis, differences in verbal memory decline between MRgLITT and traditional approaches after left‐sided resection were not statistically significant, although results suggested a tendency toward better preservation with MRgLITT. Visual memory outcomes were comparable across approaches. In contrast, MRgLITT was associated with significantly lower rates of naming decline than traditional approaches, a difference that persisted after adjusting for seizure outcomes. Although a borderline association was observed between higher seizure freedom rates and greater naming decline, this does not establish a definitive compromise in seizure outcomes. These findings suggest that, in dominant‐hemisphere surgery, particularly for patients with high language demands, MRgLITT may be a preferable option for preserving naming ability without substantially compromising seizure control. Large, prospective studies with standardized assessments and long‐term follow‐up are needed to confirm these findings and clarify the balance between seizure control and cognitive preservation.

## AUTHOR CONTRIBUTIONS

**Konstantina Stavrogianni:** Data collection; data curation; visualization; writing—original draft; writing—review & editing. **Katerina Poprelka:** Data collection; data curation; visualization; writing—review & editing. **Theodoros Fasilis:** Data collection; data curation; writing—review & editing. **Vasileios Giannopapas:** Data curation; data interpretation; writing—review & editing. **Wiebke Hahn:** Data curation; data interpretation; writing—review & editing. **Iris Gorny:** Data interpretation; writing—review & editing. **Aristotelis Kalyvas:** Data interpretation; writing—review & editing. **Lampis Stavrinou:** Data interpretation; writing—review & editing. **Sotirios Giannopoulos:** Data interpretation; writing—review & editing. **Efstathios Boviatsis:** Data interpretation; writing—review & editing. **Georgios Tsivgoulis:** Data interpretation; writing—review & editing. **Anastasios Bonakis:** Data curation; data interpretation; writing—review & editing. **Susanne Knake:** Data interpretation; writing—review & editing. **Panagiota‐Eleni Tsalouchidou:** Conceptualization; methodology; data collection; data curation; visualization; formal analysis; writing—original draft; writing—review & editing.

## CONFLICT OF INTEREST STATEMENT

A.B. has received honoraria from Angelini, UCB, and Swiss Bio Pharma, none of which are related to the present work. S.K. has received speaker's honoraria from Bial, Destin, Jazz Pharma, Merck Serono, and UCB, none of which are related to the present work. P.‐E.T. has received research grants from the German Society for Epileptology (Otfrid‐Foerster Stipendium), as well as travel grants and honoraria for lectures from UCB and Angelini, none of which are related to the present work. None of the other authors has any conflict of interest to disclose.

## ETHICS STATEMENT

Ethical approval was not required, as this study is based exclusively on previously published data. We confirm that we have read the Journal's position on issues involved in ethical publication and affirm that this report is consistent with those guidelines.

## Supporting information


Data S1.

## Data Availability

All data relevant to this study are included in the article and supplementary materials. Additional data may be made available upon request from the corresponding author.
