# Supplementary material for: Neuropsychological outcomes comparing traditional surgical approaches and laser interstitial thermal therapy for refractory mesial temporal lobe epilepsy: A systematic review and meta‐analysis
Source: Epilepsia. 2025 Oct 18;67(2):588–605. doi: 10.1111/epi.18687 (PMC12927677; doi:10.1111/epi.18687)
Supplement: Supplementary file 1 — Data S1. [file EPI-67-588-s001.docx]

**Supplementary Material**

**S1. Search Strategy**

**MEDLINE (via PubMed)**

1. (((((((((selective amygdalohippocampectomy) OR (SAH)) OR (SAHE)) OR (anterior temporal lobectomy)) OR (temporal lobectomy)) OR (ATLR)) OR (ATL)) AND (epilepsy)) AND (drug resistant)) AND (focal)
2. (((TLE) OR (temporal lobe epilepsy)) AND (LITT)) OR (Laser interstitial thermal therapy)

**EMBASE**

1. ('selective amygdalohippocampectomy'/exp OR 'selective amygdalohippocampectomy' OR SAH OR SAHE OR 'anterior temporal lobectomy'/exp OR 'anterior temporal lobectomy' OR 'temporal lobectomy'/exp OR 'temporal lobectomy' OR ATLR OR ATL) AND ('epilepsy'/exp OR epilepsy) AND ('drug resistant epilepsy'/exp OR 'drug resistant') AND ('focal epilepsy'/exp OR focal)
2. ('tle' OR 'temporal lobe epilepsy'/exp OR 'temporal lobe epilepsy') AND ('litt' OR 'laser interstitial thermal therapy'/exp OR 'laser interstitial thermal therapy')

**Scopus**

1. TITLE-ABS-KEY("selective amygdalohippocampectomya" OR "selective amygdalohippocampectomy" OR SAH OR SAHE OR "anterior temporal lobectomy" OR "temporal lobectomy" OR ATLR OR ATL) AND TITLE-ABS-KEY("epilepsy" OR epilepsy) AND TITLE-ABS-KEY("drug resistant epilepsy" OR "drug resistant") AND TITLE-ABS-KEY("focal epilepsy" OR focal)
2. (((tle) OR (temporal AND lobe AND epilepsy)) AND (litt)) OR (laser AND interstitial AND thermal AND therapy)

**S2. Risk of Bias Assessment**

**Figure S2.1** Summary of risk of bias assessment for studies reporting neuropsychological outcomes following traditional surgical treatments


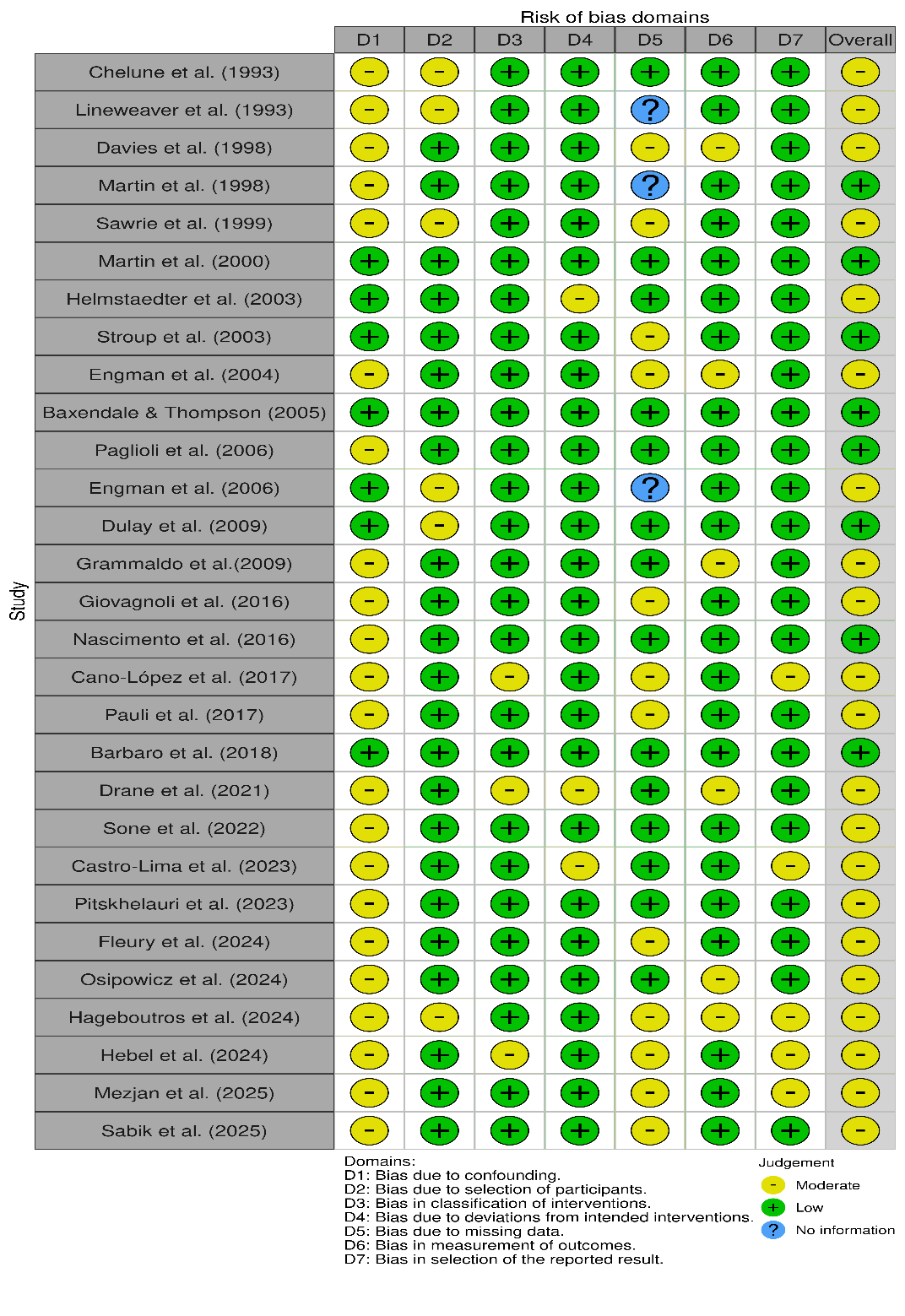


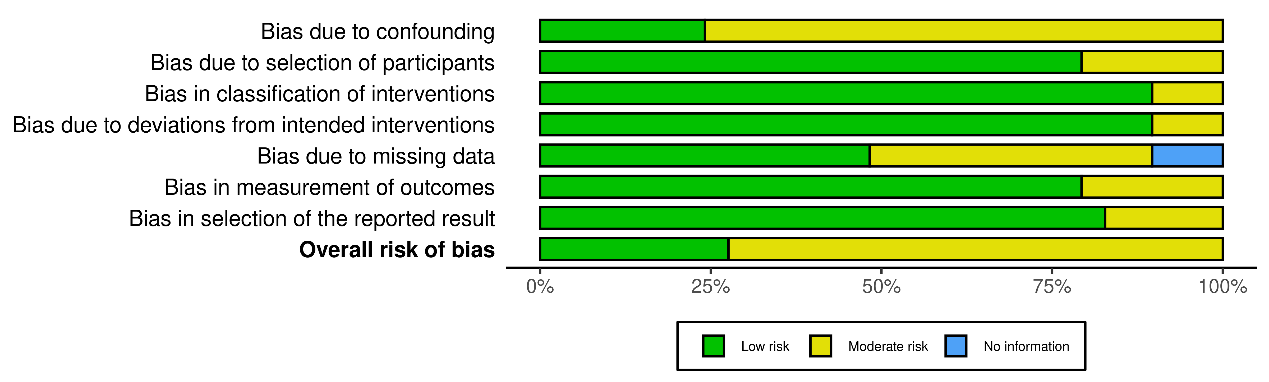


**Figure S2.2.:** Summary of risk of bias assessment for studies reporting neuropsychological outcomes after MRgLITT for temporal lobe epilepsy.


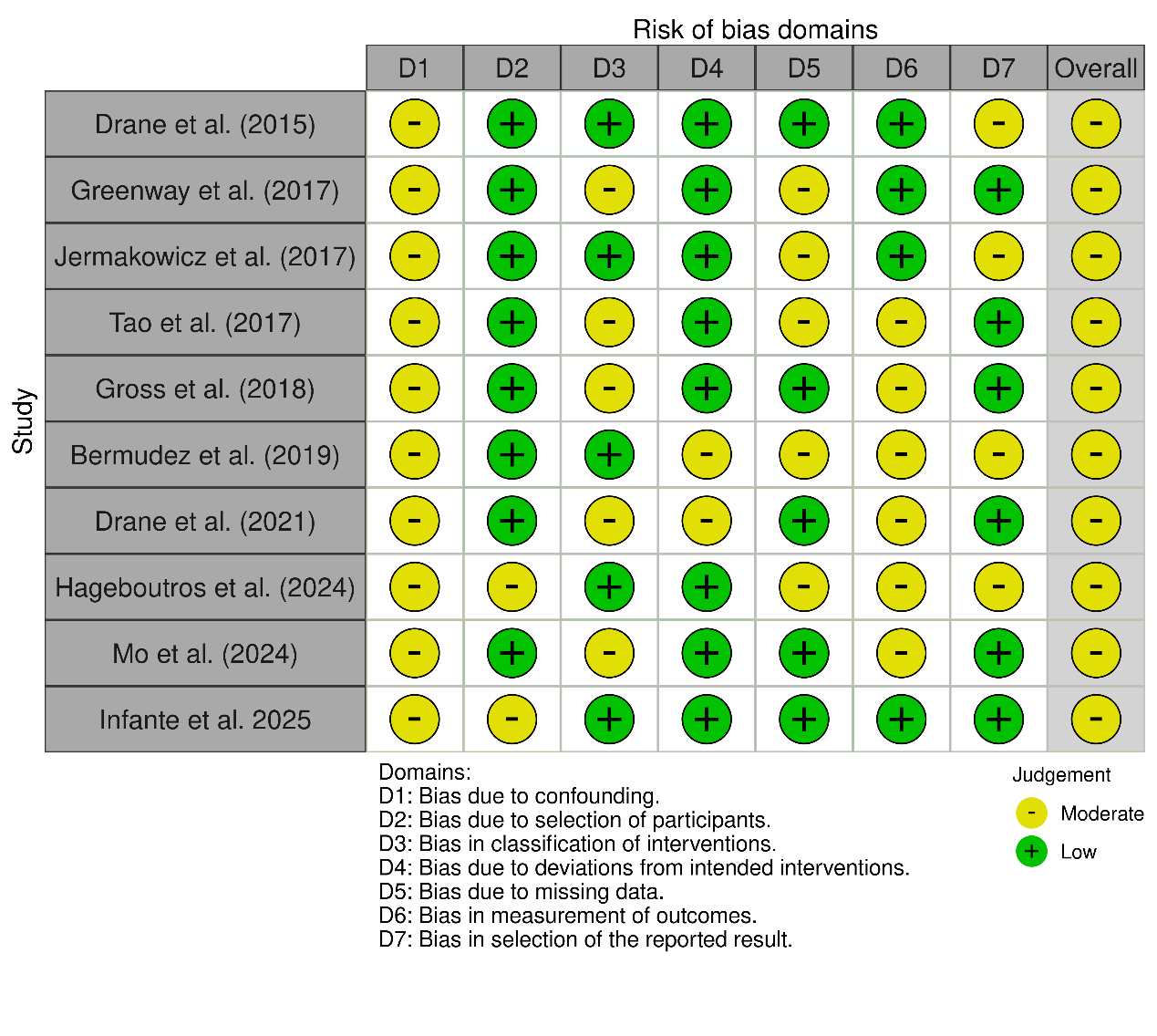


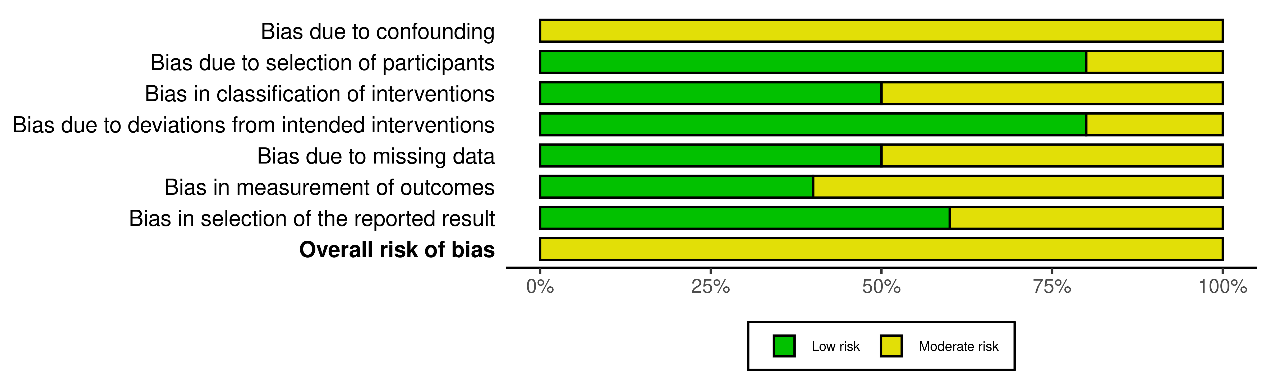


**S3. Forest Plots of Neuropsychological Outcomes Following Traditional Surgery Approaches**

**S3.1. Verbal Memory**


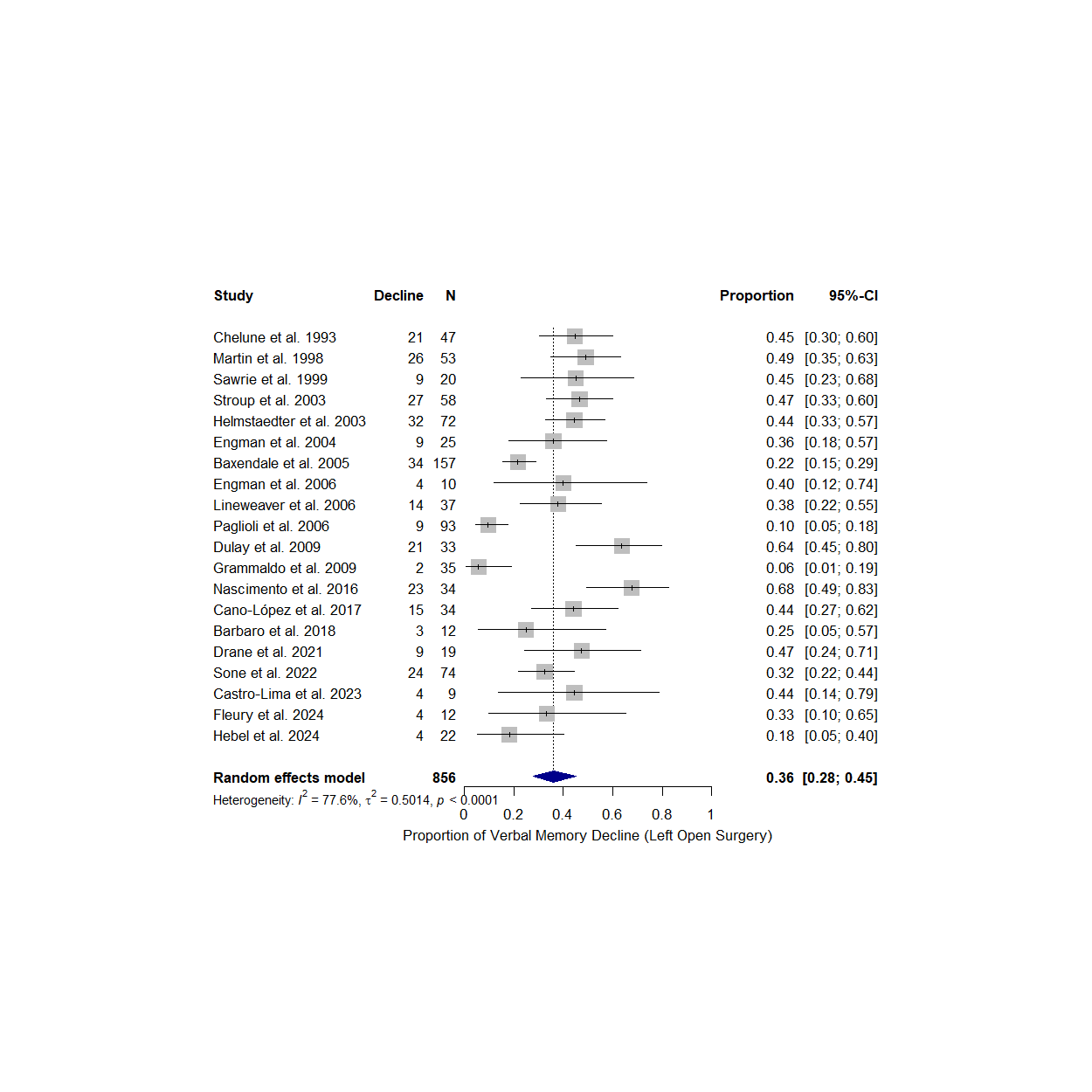


**Figure S3.1.1.:** Forest plot showing the proportion of verbal memory decline following left-sided open surgery


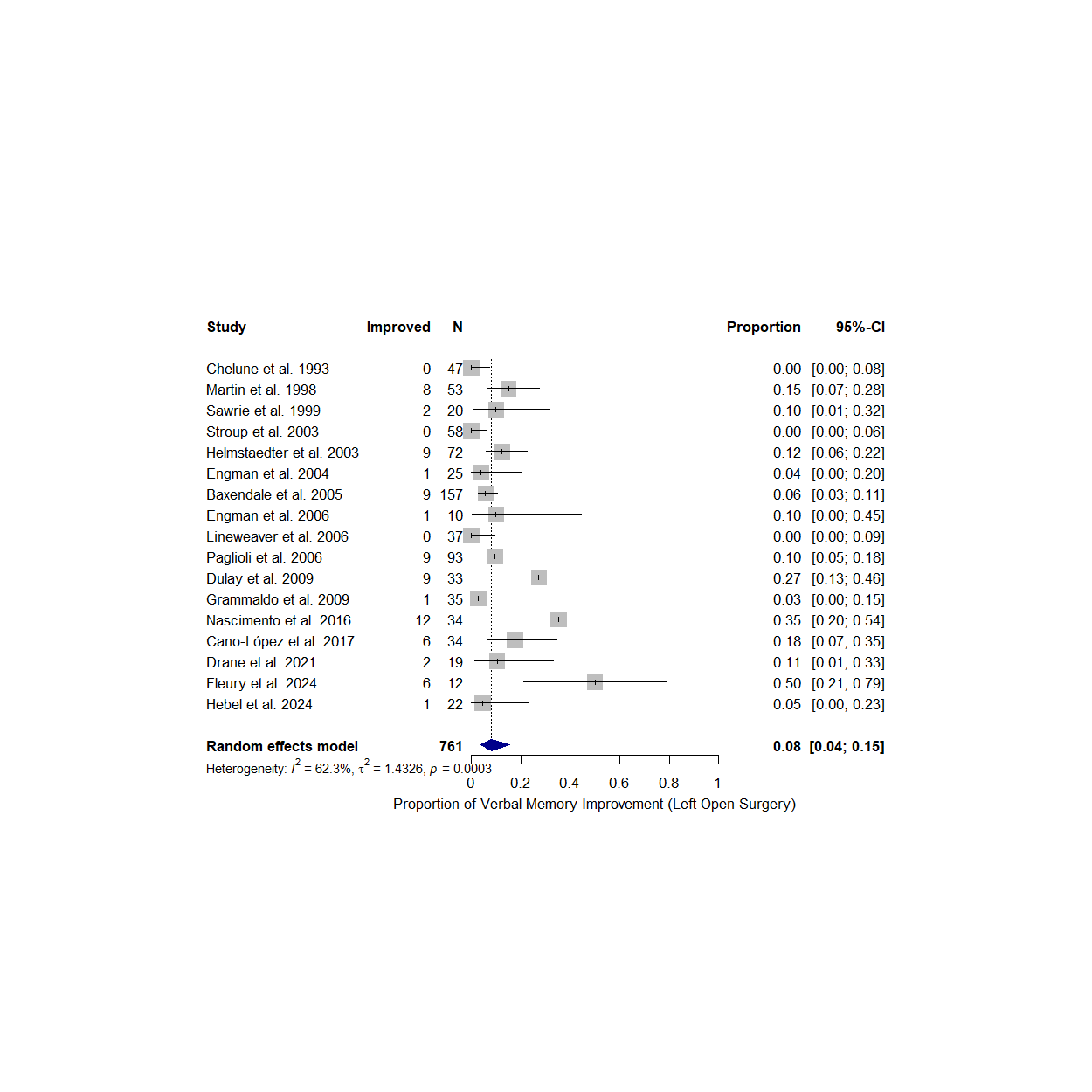


**Figure S3.1.2.:** Forest plot showing the proportion of verbal memory improvement following left-sided open surgery


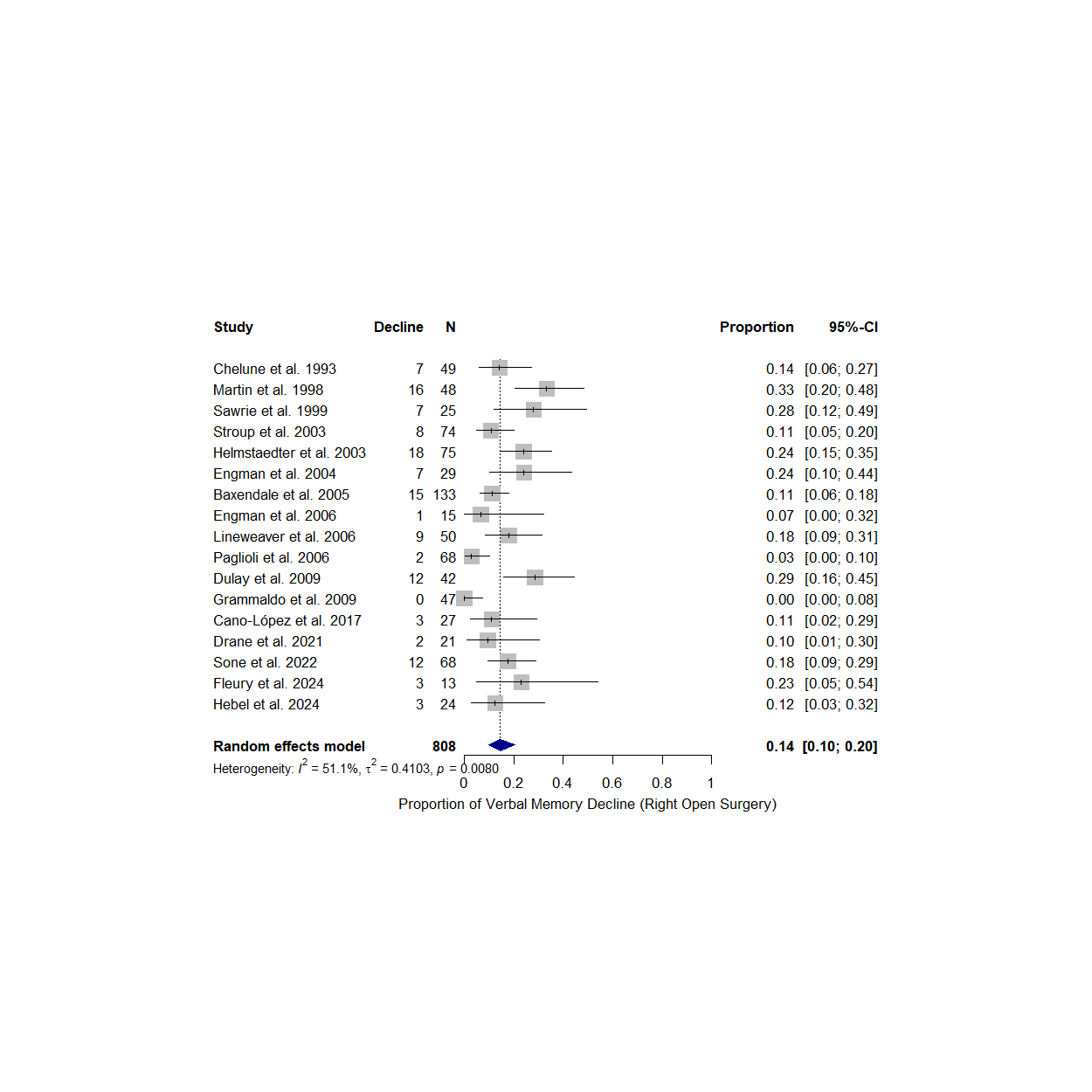


**Figure S3.1.3.:** Forest plot showing the proportion of verbal memory decline following right-sided open surgery


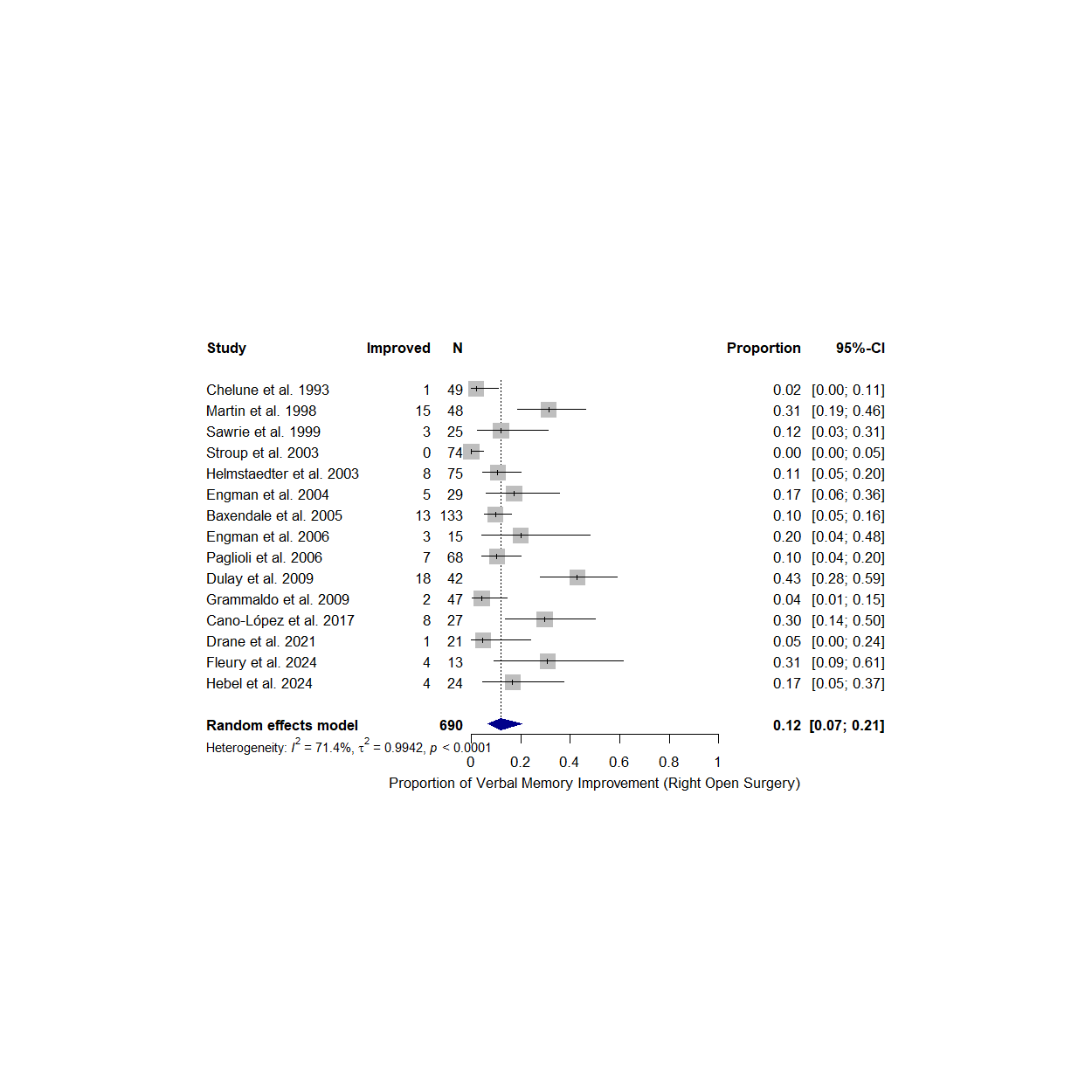


**Figure S3.1.4:** Forest plot showing the proportion of verbal memory improvement following right-sided open surgery

**S3.2. Visual Memory**

**
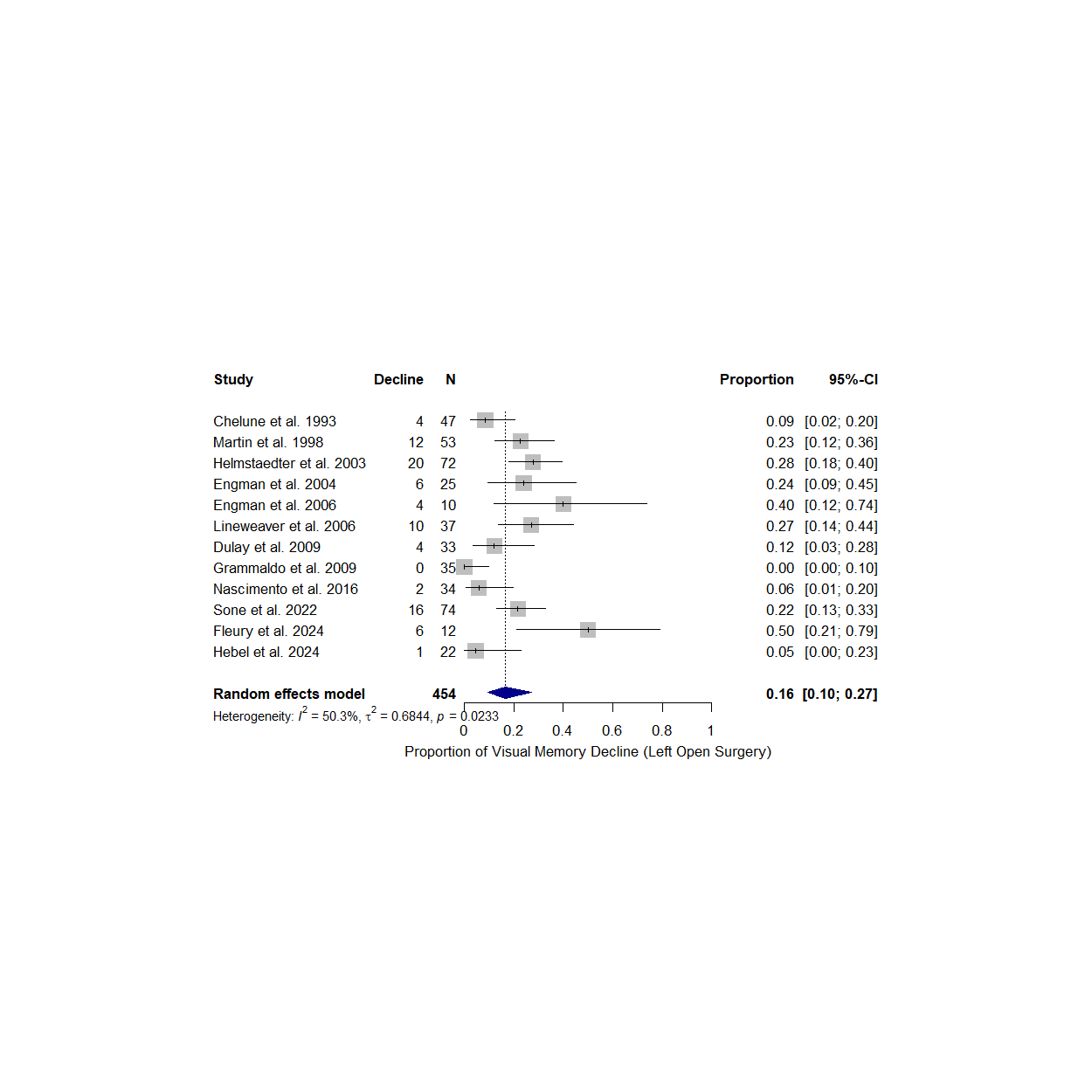
**

**Figure S3.2.1.:** Forest plot showing the proportion of visual memory decline following left-sided open surgery

**
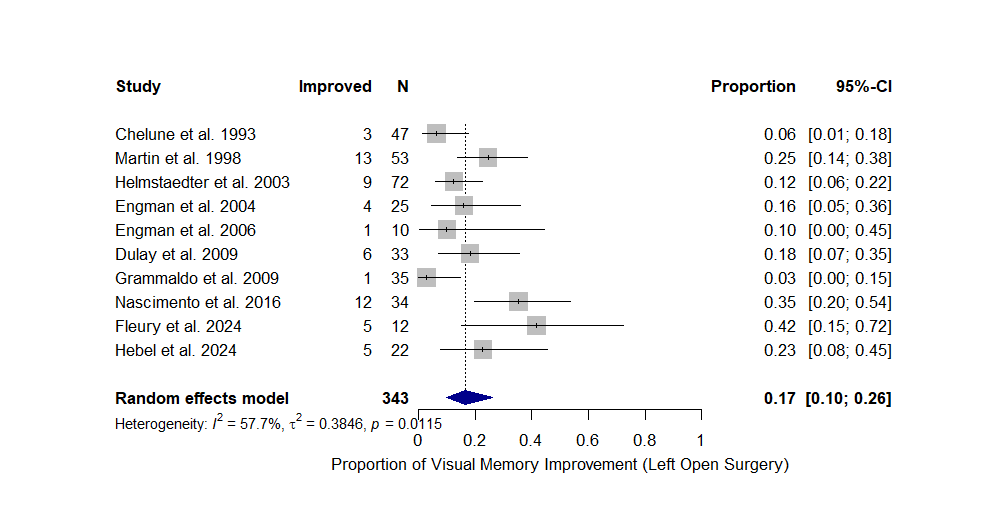
**

**Figure S3.2.2.:** Forest plot showing the proportion of visual memory improvement following left-sided open surgery

**
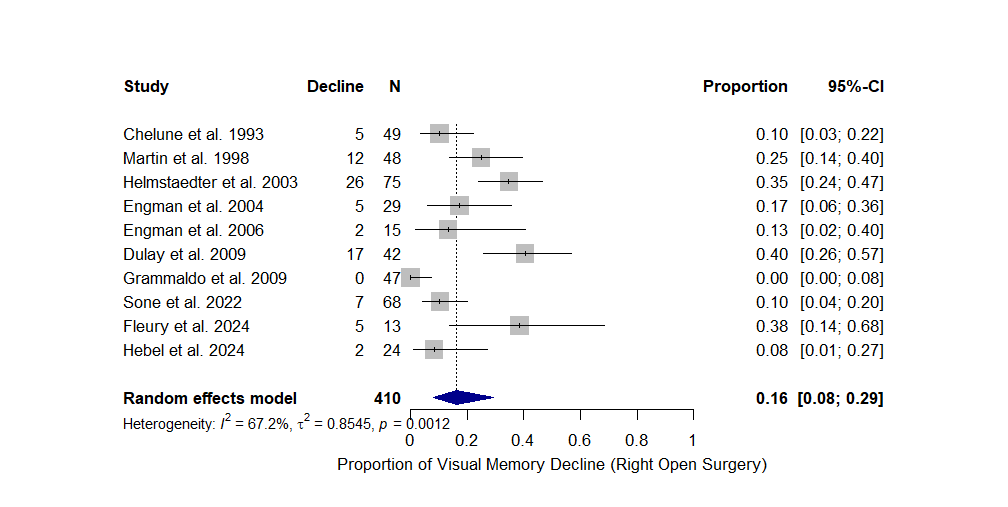
**

**Figure S3.2.3.:** Forest plot showing the proportion of visual memory decline following right-sided open surgery

**
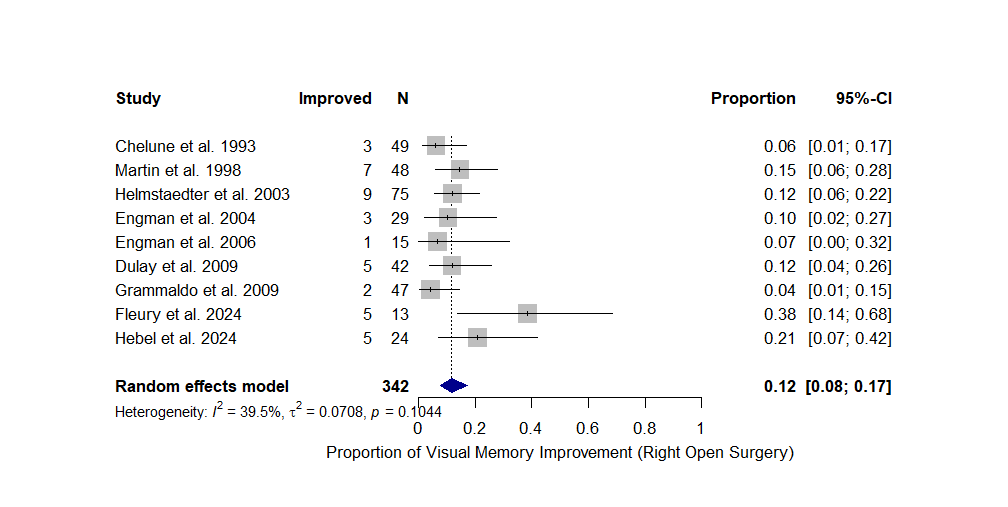
**

**Figure S3.2.4.:** Forest plot showing the proportion of visual memory improvement following right-sided open surgery


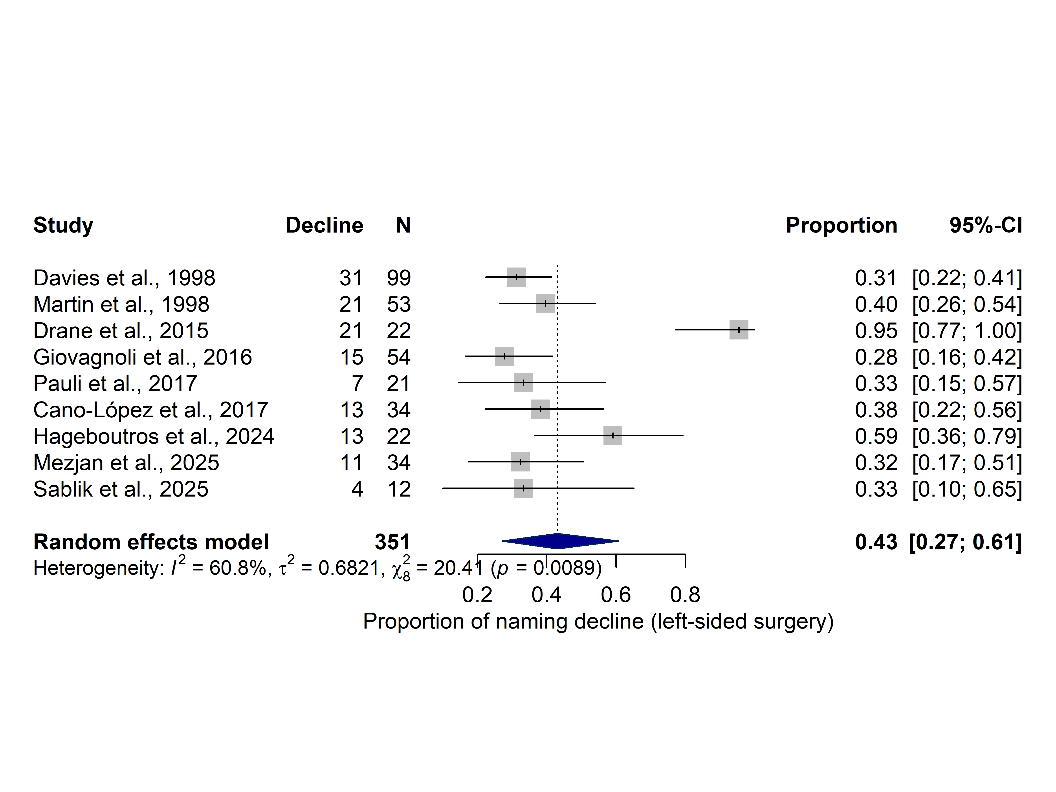
**S3.3. Naming**

**Figure S3.3.1.:** Forest plot showing the proportion of naming decline following left-sided open surgery

**
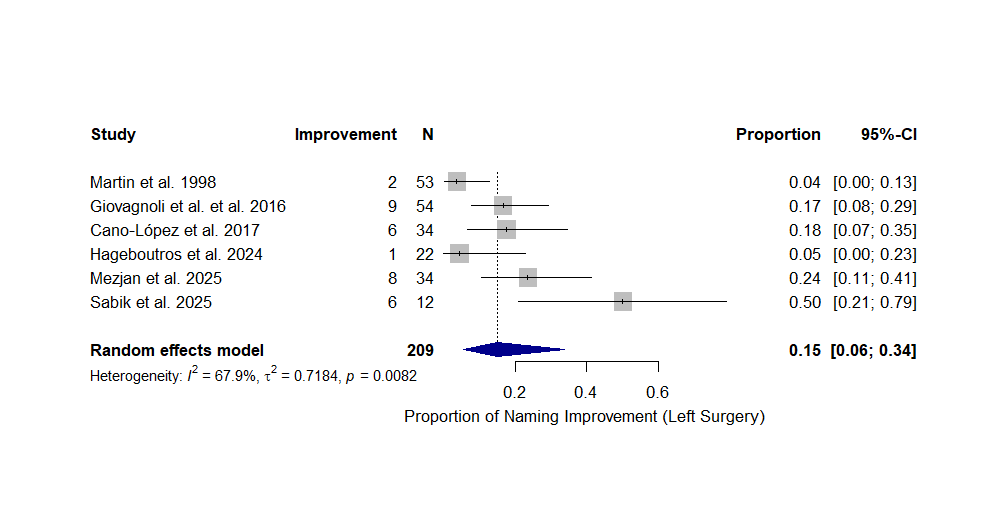
**

**Figure S3.3.2.**: Forest plot showing the proportion of naming improvement following left-sided open surgery

**
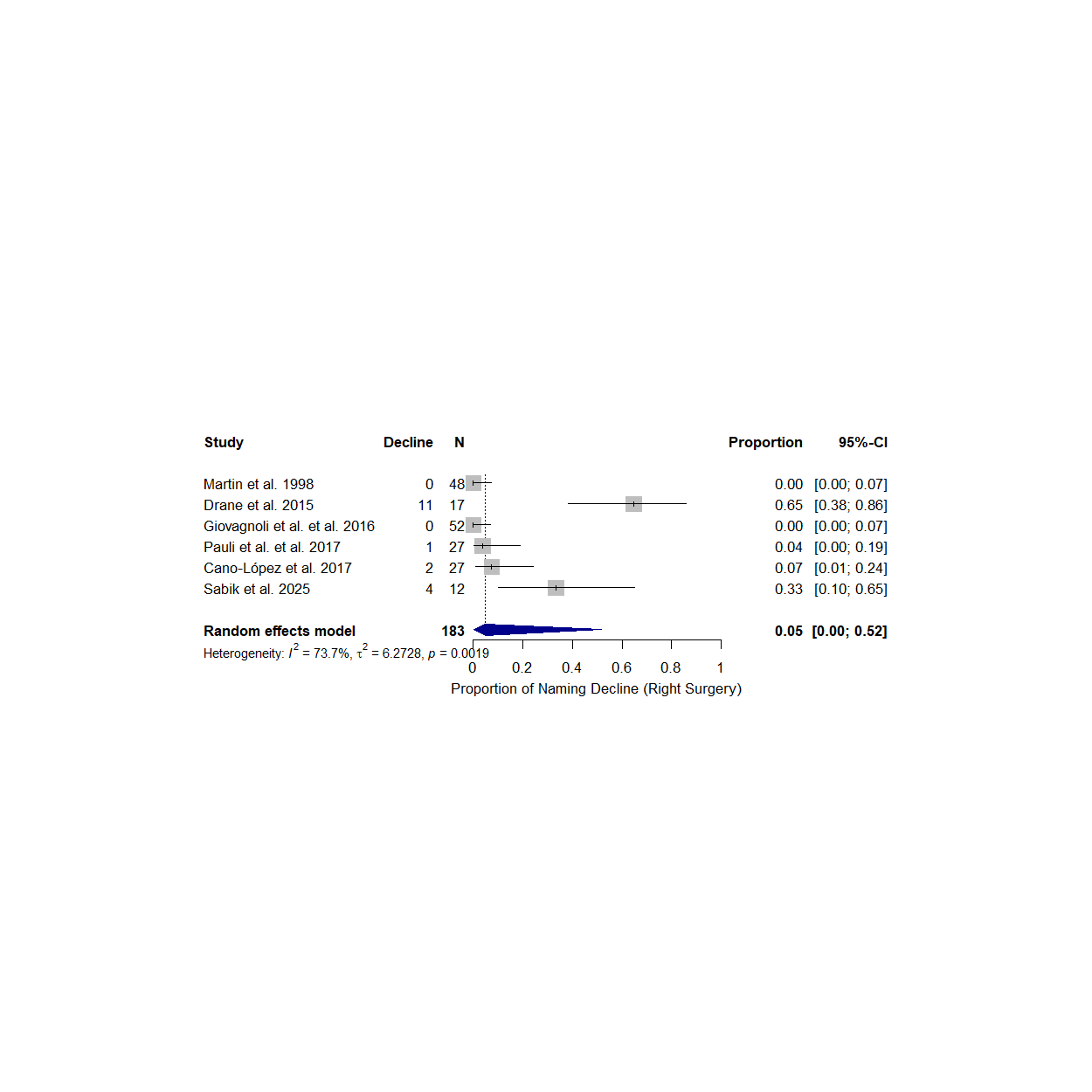
**

**Figure S3.3.3.**: Forest plot showing the proportion of naming decline following right-sided open surgery

**
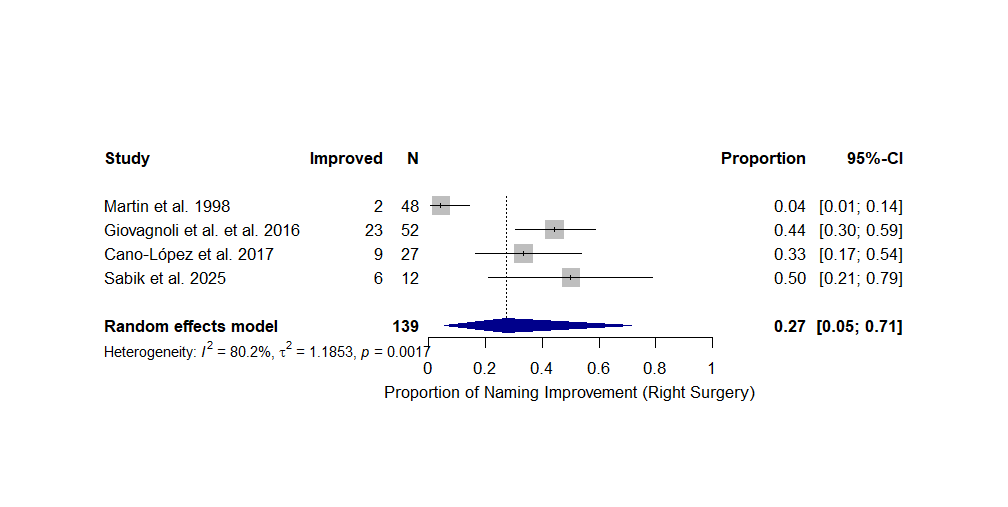
**

**Figure S3.3.4.:** Forest plot showing the proportion of naming improvement following right-sided open surgery

**S4. Forest Plots of Neuropsychological Outcomes Following MR-guided Laser Interstitial Thermal Therapy (MRgLITT)**

**S4.1. Verbal Memory**

**
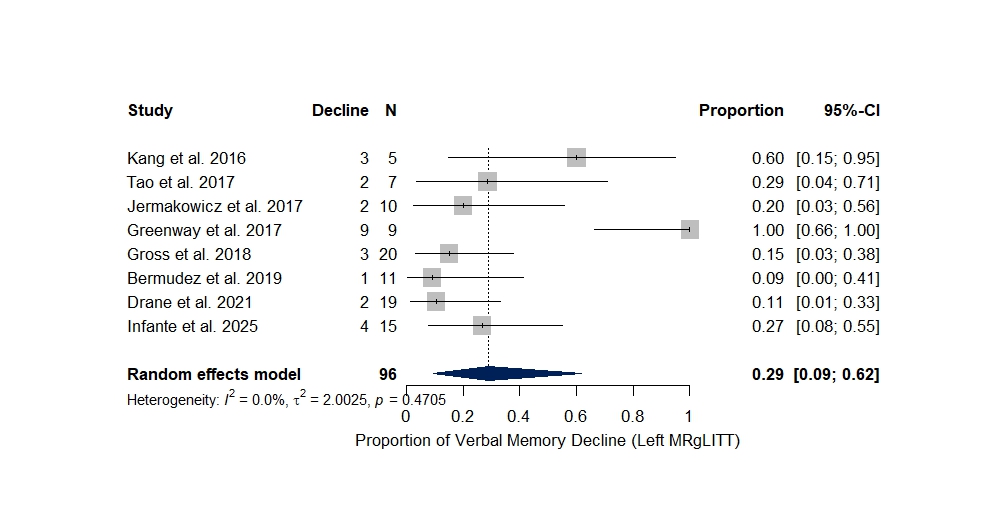
**

Figure S4.1.1: Forest plot showing the proportion of verbal memory decline after left-sided MR-guided Laser Interstitial Thermal Therapy.

**
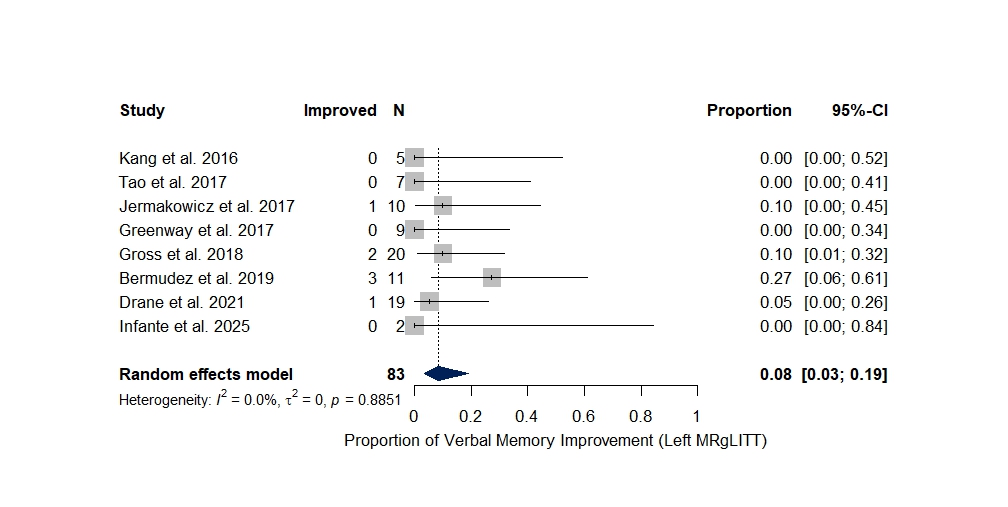
**

Figure S4.1.2: Forest plot showing the proportion of verbal memory improvement after left-sided MR-guided Laser Interstitial Thermal Therapy.


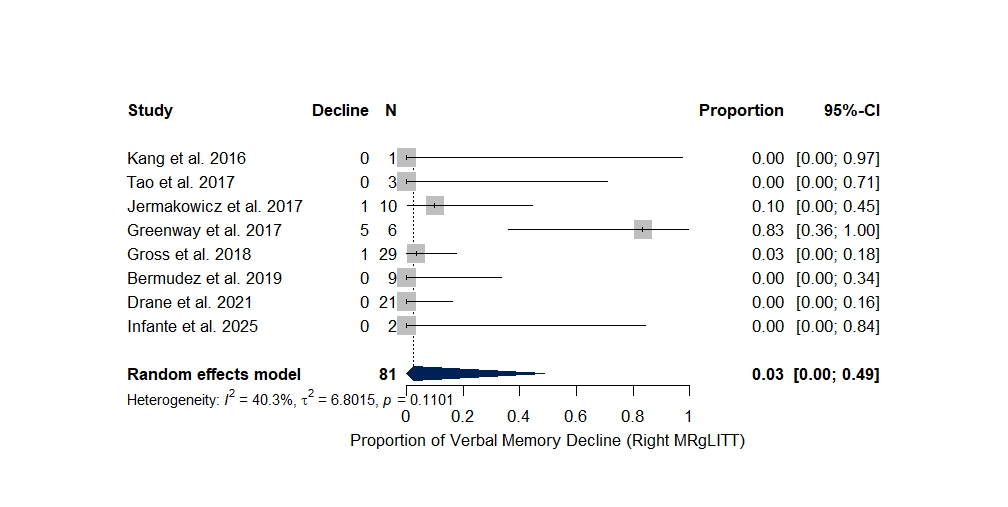


Figure S4.1.3.: Forest plot showing the proportion of verbal memory decline after right-sided MR-guided Laser Interstitial Thermal Therapy.

**
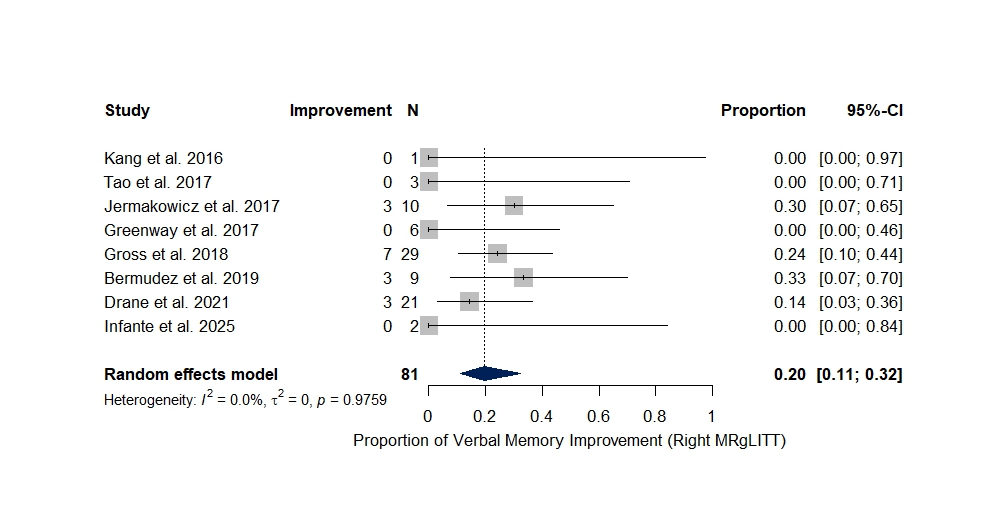
**

Figure S4.1.4.: Forest plot showing the proportion of verbal memory improvement after right-sided MR-guided Laser Interstitial Thermal Therapy.

**S4.2. Visual Memory**

**
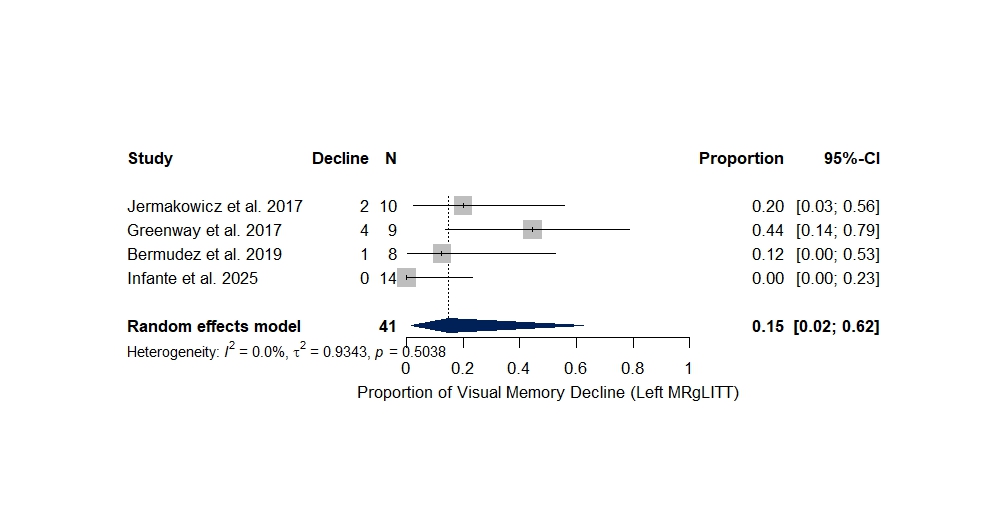
**

Figure S4.2.1.: Forest plot showing the proportion of visual memory decline after left-sided MR-guided Laser Interstitial Thermal Therapy.

**
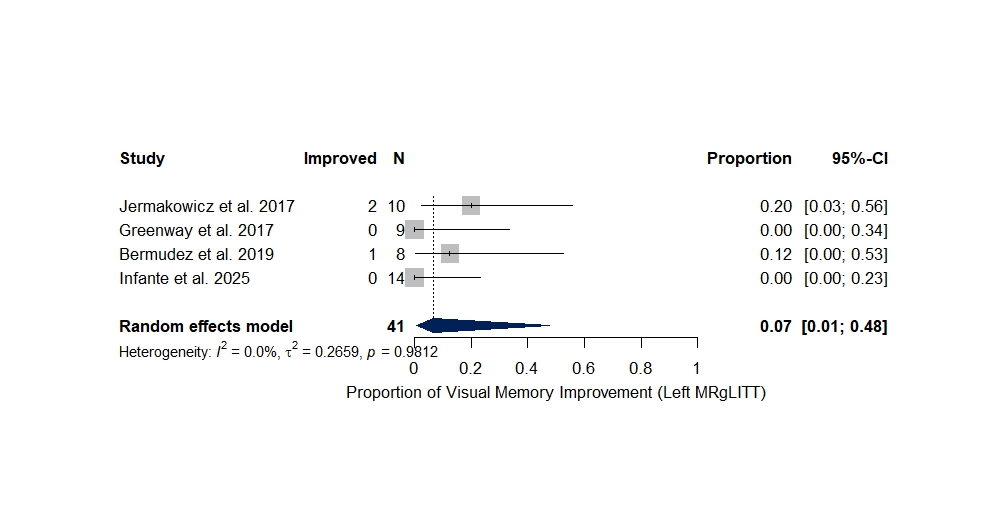
**

Figure S4.2.2.: Forest plot showing the proportion of visual memory improvement after left-sided MR-guided Laser Interstitial Thermal Therapy.

**
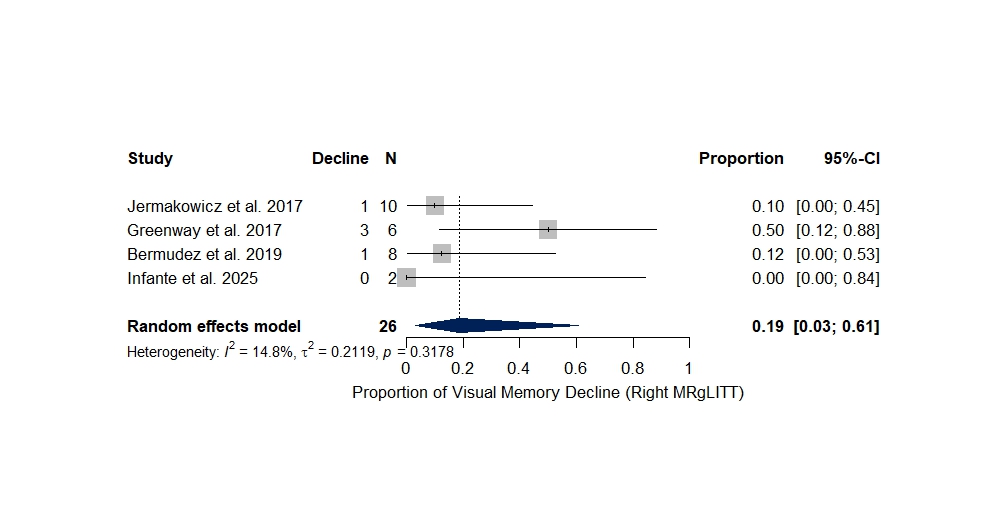
**

Figure S4.2.3.: Forest plot showing the proportion of visual memory decline after right-sided MR-guided Laser Interstitial Thermal Therapy.

**
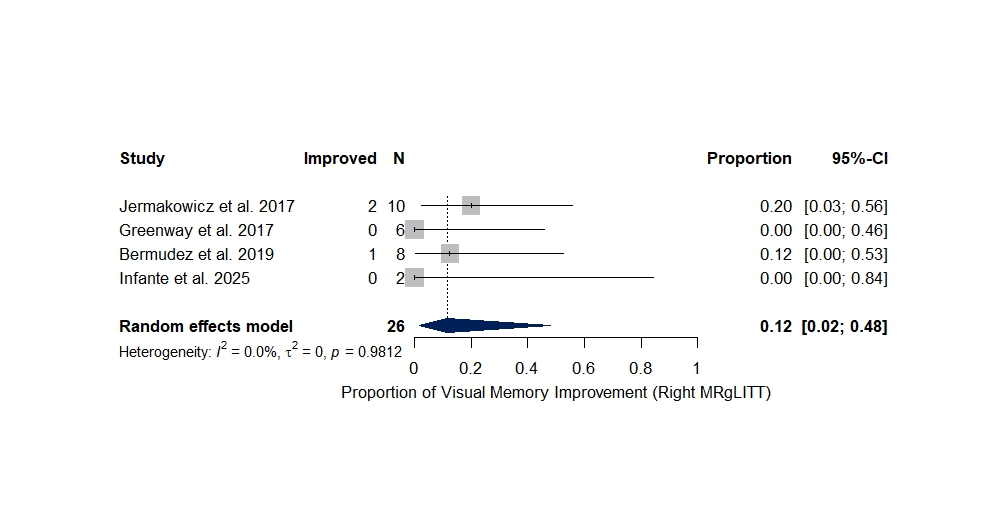
**

Figure S4.2.4: Forest plot showing the proportion of visual memory improvement after right-sided MR-guided Laser Interstitial Thermal Therapy

**S4.3. Naming**

**
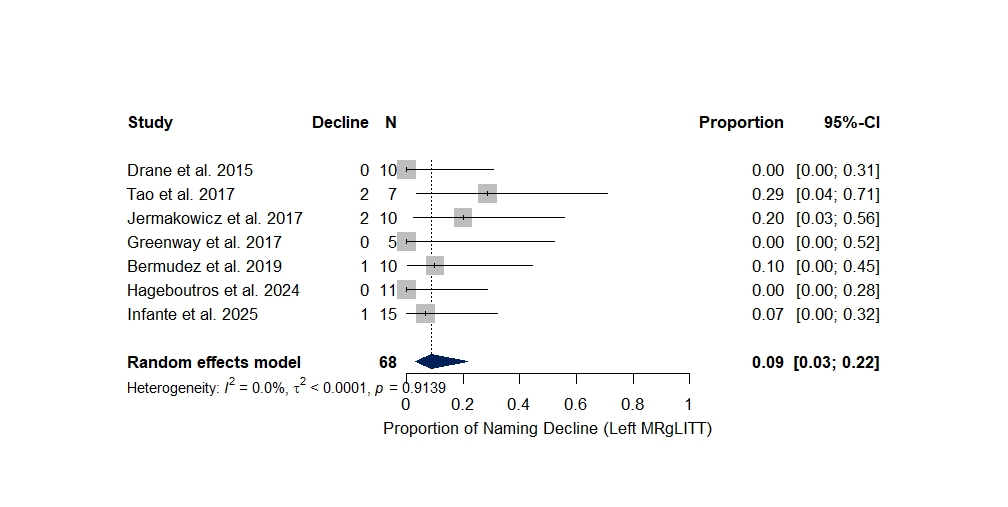
**

Figure S4.3.1.: Forest plot showing the proportion of naming decline after left-sided MR-guided Laser Interstitial Thermal Therapy.

**
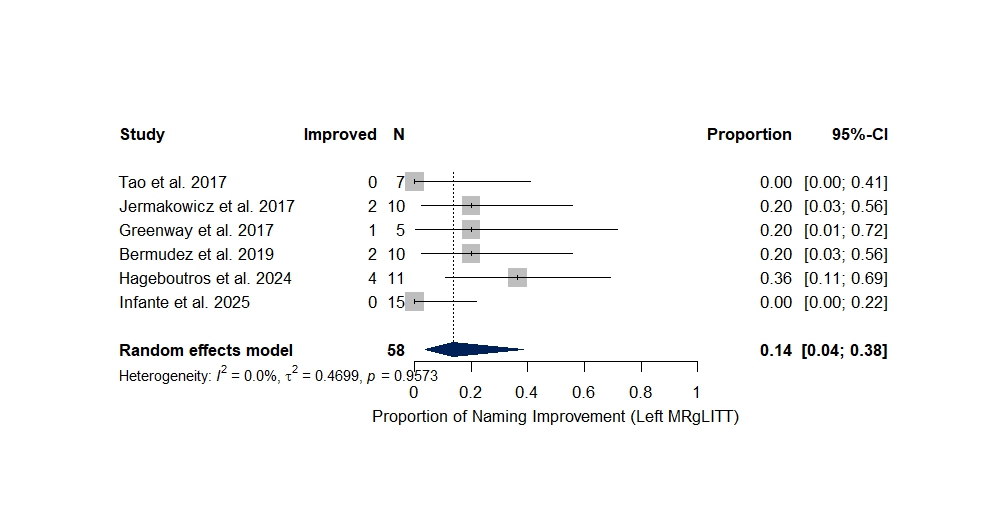
**

Figure S4.3.2.: Forest plot showing the proportion of naming improvement after left-sided MR-guided Laser Interstitial Thermal Therapy.

**
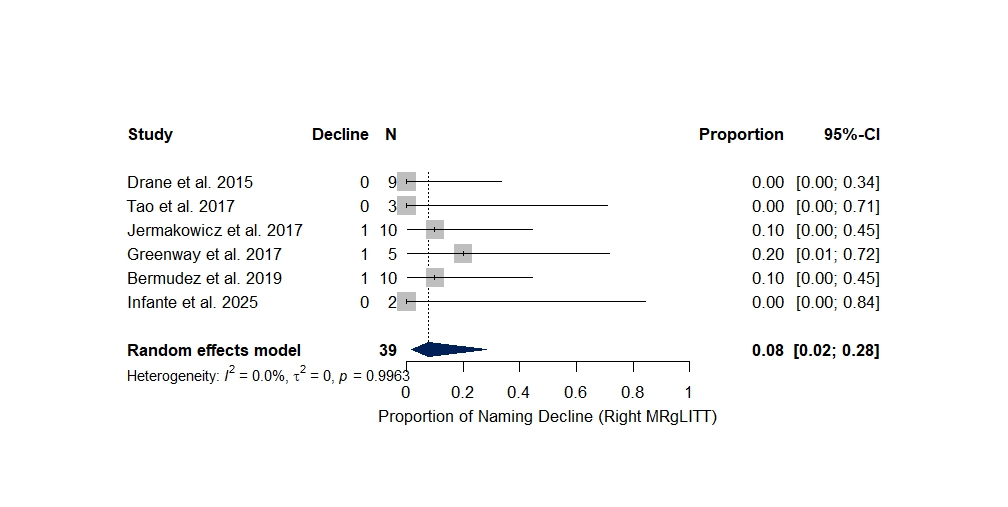
**

Figure S4.3.3.: Forest plot showing the proportion of naming decline after right-sided MR-guided Laser Interstitial Thermal Therapy.

**
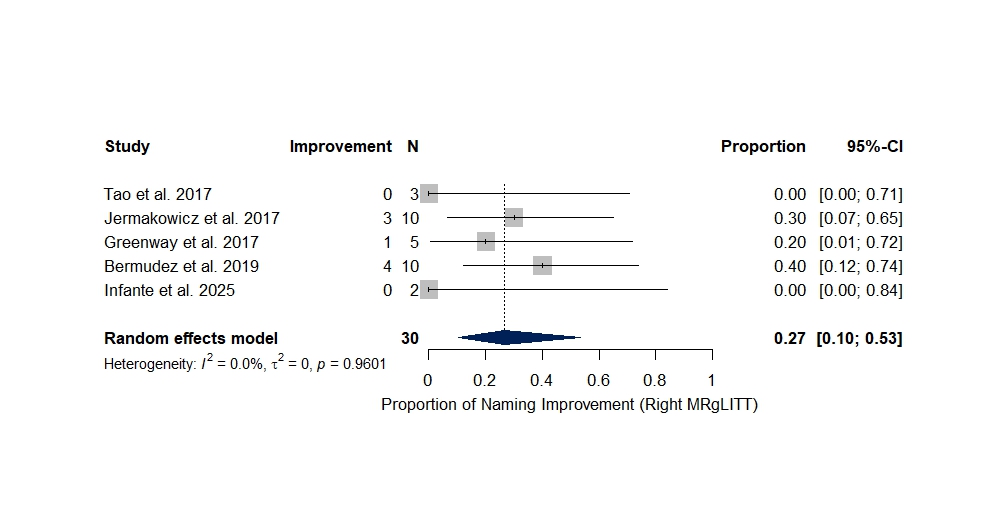
**

Figure S4.3.4.: Forest plot showing the proportion of naming improvement after right-sided MR-guided Laser Interstitial Thermal Therapy.

**Table S5.1.: Meta-Regression Results: Verbal Memory Decline (Left-Sided Procedures)**

| Term | Estimate | SE | z-value | p-value | 95% CI Lower | 95% CI Upper |
| --- | --- | --- | --- | --- | --- | --- |
| Intercept (MRgLITT) | -0.5364 | 0.1702 | -3.1512 | 0.0016 | -0.8700 | -0.2028 |
| Surgery (vs MRgLITT) | -0.5419 | 0.4057 | -1.3355 | 0.1817 | -1.3371 | 0.2533 |

Model diagnostics: Test of Moderators: QM(df = 1) = 1.78, p = 0.1817; Residual heterogeneity: τ² = 0.4058, I² = 72.40%, R² = 12.43%; Test for residual heterogeneity: QE(df = 26) = 99.72, p < 0.0001; Model fit: AIC = 80.56Note: The intercept represents the log-proportion of verbal memory decline in the MRgLITT group. The surgery coefficient compares open resection to MRgLITT. Estimates are reported on the log-proportion scale.

Table S5.2. Meta-Regression Results: Visual Memory Decline (Right-Sided Procedures)

| Term | Estimate | SE | z.value | p.value | 95% CI Lower | 95% CI Upper |
| --- | --- | --- | --- | --- | --- | --- |
| Intercept (MRgLITT) | -1.2629 | 0.6191 | -2.0397 | 0.04138 | -2.4764 | -0.0494 |
| Surgery (vs MRgLITT) | -0.1459 | 0.6708 | -0.2175 | 0.8278 | -1.4606 | 1.1688 |

Model diagnostics: Test of Moderators: QM(df = 1) = 0.05, p = 0.8278; Residual heterogeneity: τ² = 0.3978, I² = 62.86%, R² = 0%; Test for residual heterogeneity: QE(df = 12) = 36.87, p = 0.0002342; Model fit: AIC = 45.08. Note: The intercept represents the log-proportion of visual memory decline in the MRgLITT group. The surgery coefficient compares open resection to MRgLITT. Estimates are reported on the log-proportion scale.

Table S5.3. Meta-Regression Results: Naming Decline (Left-Sided Procedures)

| Term | Estimate | SE | z.value | p.value | 95% CI Lower | 95% CI Upper |
| --- | --- | --- | --- | --- | --- | --- |
| Intercept (MRgLITT) | -1.9195 | 0.4022 | -4.7728 | < 0.0001 | -2.7077 | -1.1312 |
| Surgery (vs MRgLITT) | 1.3674 | 0.4213 | 3.2458 | 0.0012 | 0.5417 | 2.1931 |

Model diagnostics: Test of Moderators: QM(df = 1) = 10.54, p = 0.0012; Residual heterogeneity: τ² = 0.0137, I² = 6.15%, R² = 0.00%; Test for residual heterogeneity: QE(df = 14) = 24.28, p = 0.0424. Note: The intercept represents the log-proportion of naming decline in the MRgLITT group. The surgery coefficient compares open resection to MRgLITT. Estimates are reported on the log-proportion scale.

Table S5.4. Meta-regression results of naming decline and seizure freedom

| Term | Estimate | Std. Error | Z value | P value | CI Lower | CI Upper |
| --- | --- | --- | --- | --- | --- | --- |
| intercept | -0.5652 | 0.1660 | -3.4048 | 0.0007 | -0.8905 | -0.2398 |
| seizure_freedom | 0.0146 | 0.0075 | 1.9535 | 0.0508 | -0.0000 | 0.0293 |
| surgery_type | -1.3589 | 0.4314 | -3.1498 | 0.0016 | -2.2045 | -0.5133 |

Model diagnostics: Test of Moderators: QM(df = 2) = 15.54, *p* = 0.0004; Residual heterogeneity: τ² = 0.0081, I² = 2.75%, R² = 98.96%; Test for residual heterogeneity: QE(df = 11) = 18.59, *p* = 0.0688; Model fit: AIC = 40.31, BIC = 41.90; Note: The intercept represents the log-proportion of naming decline in the open surgery group. The surgery coefficient compares MRgLITT to open resection. Estimates are reported on the log-proportion (logit) scale.

Table S5.5. Meta-regression results including interaction term

| Term | Estimate | Std. Error | Z value | P value | CI Lower | CI Upper |
| --- | --- | --- | --- | --- | --- | --- |
| intercept | -0.5652 | 0.1660 | -3.4048 | 0.0007 | -0.8905 | -0.2398 |
| seizure_freedom | 0.0146 | 0.0076 | 1.9097 | 0.0562 | -0.0004 | 0.0296 |
| surgery_type | -0.2101 | 2.1859 | -0.0961 | 0.9234 | -4.4945 | 4.0742 |
| seizure_freedom:surgery_type | -1.6325 | 3.0280 | -0.5391 | 0.5898 | -7.5672 | 4.3023 |

Model diagnostics: Test of Moderators: QM(df = 3) = 15.63, *p* = 0.0014; Residual heterogeneity: τ² = 0.0151, I² = 5.28%, R² = 98.08%; Test for residual heterogeneity: QE(df = 10) = 18.29, *p* = 0.0502; Model fit: AIC = 40.35, BIC = 41.86; Note: The intercept represents the log-proportion of naming decline in the open surgery group. The interaction term tests whether the effect of seizure freedom on naming decline differs by surgical approach.

**S6 Forest Plots of Subgroup Analyses (Language Dominance-Based Comparisons)**

S6.1. Verbal Memory Dominant Procedures


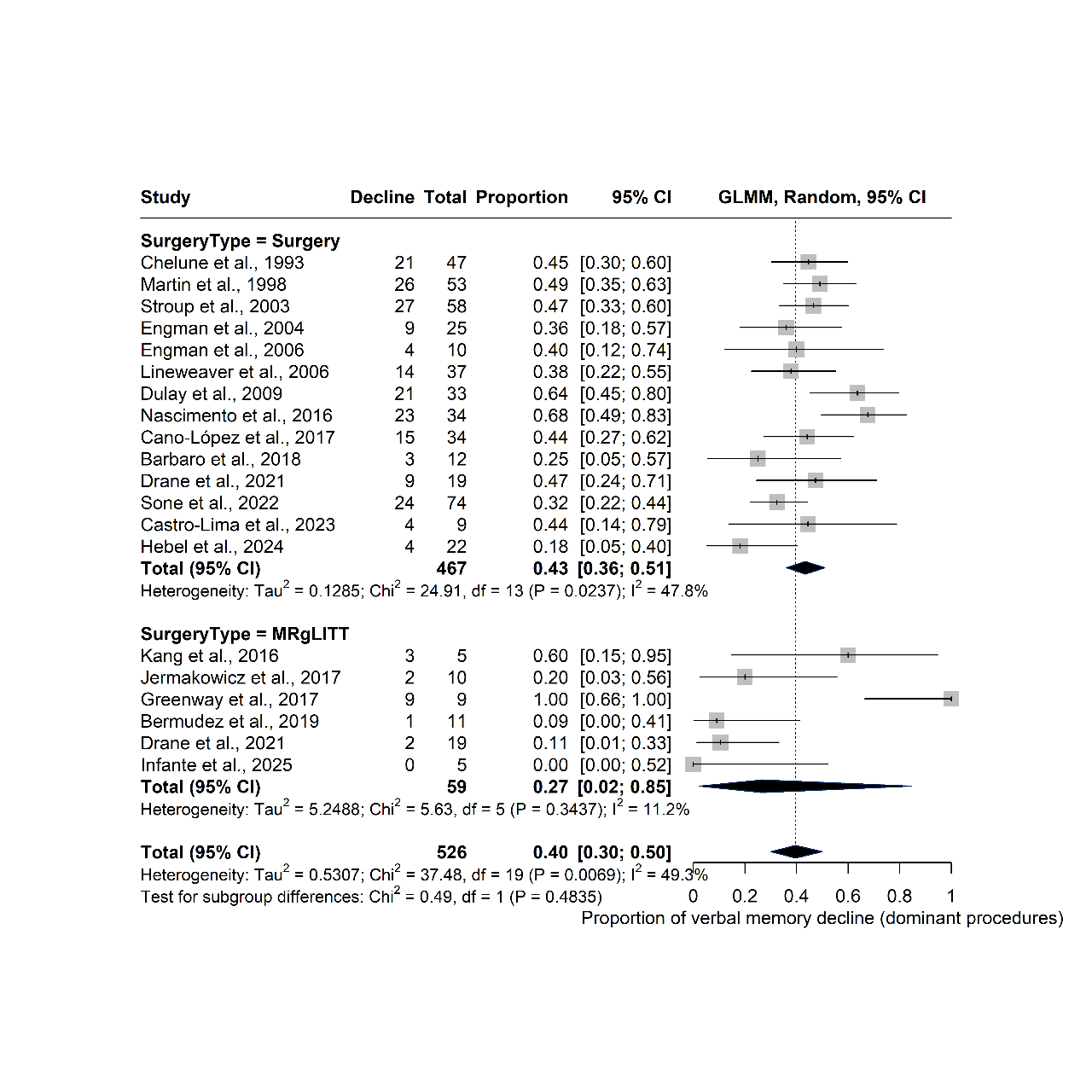


Figure S6.1.: Forest plot showing the proportion of verbal memory decline after dominant-hemisphere procedures, comparing open resection and MRgLITT.

S6.2. Visual Memory Non-Dominant Procedures


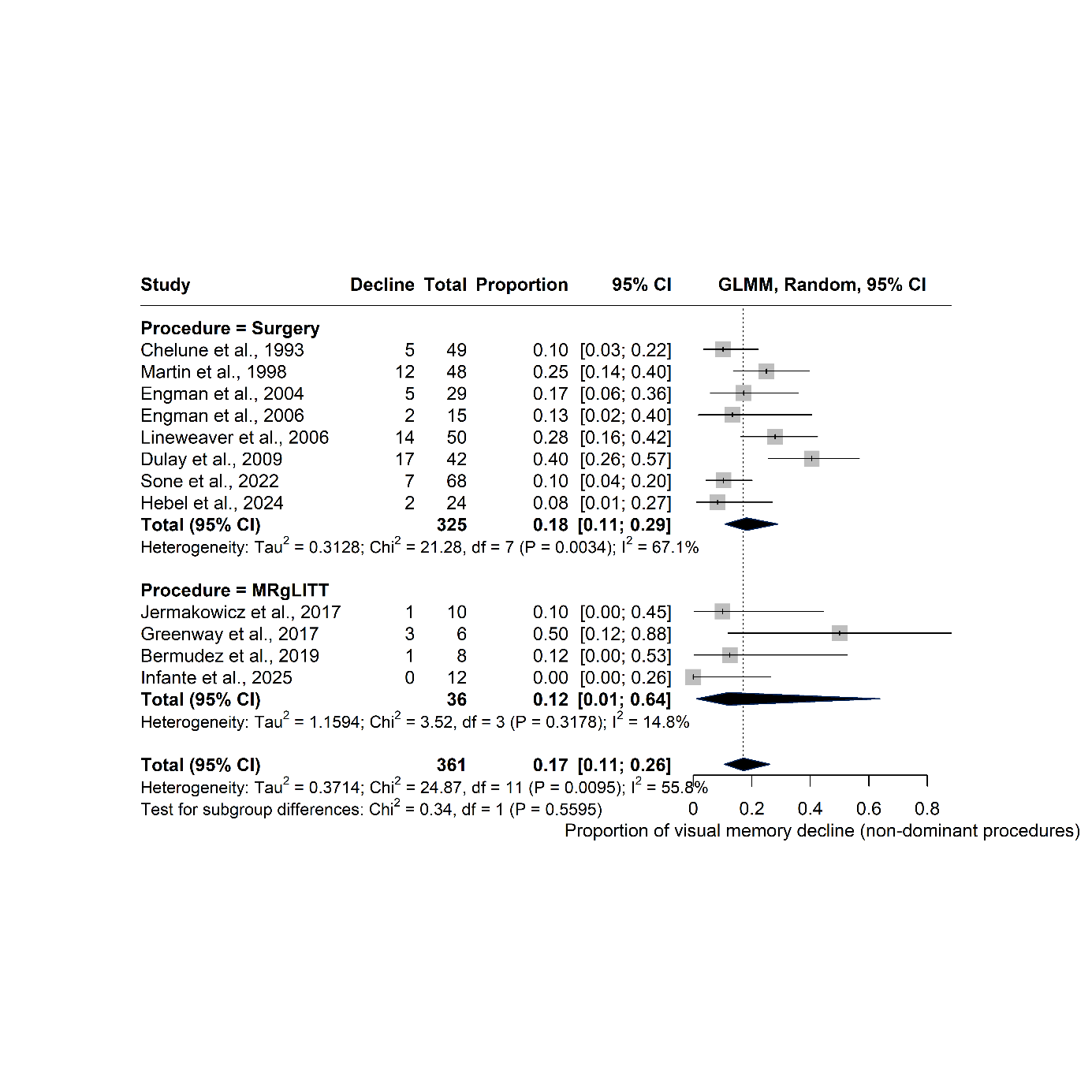


Figure S6.2.: Forest plot showing the proportion of visual memory decline after non-dominant-hemisphere procedures, comparing open resection and MRgLITT

S6.3. Naming Dominant Procedures


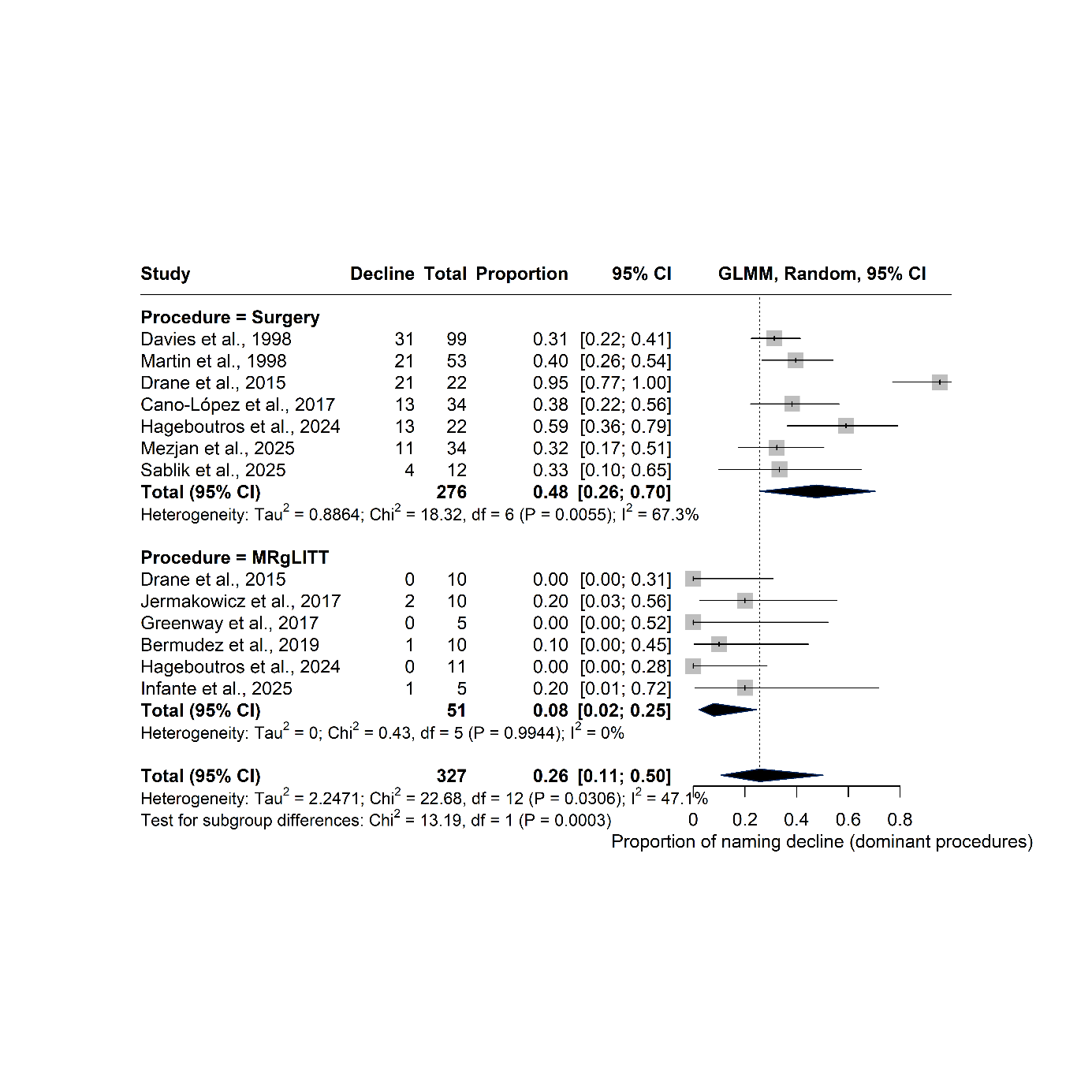


Figure S6.3.: Forest plot showing the proportion of naming decline after dominant-hemisphere procedures, comparing open resection and MRgLITT

S6.4. Subgroup Comparison of SAHE and MRgLITT

***
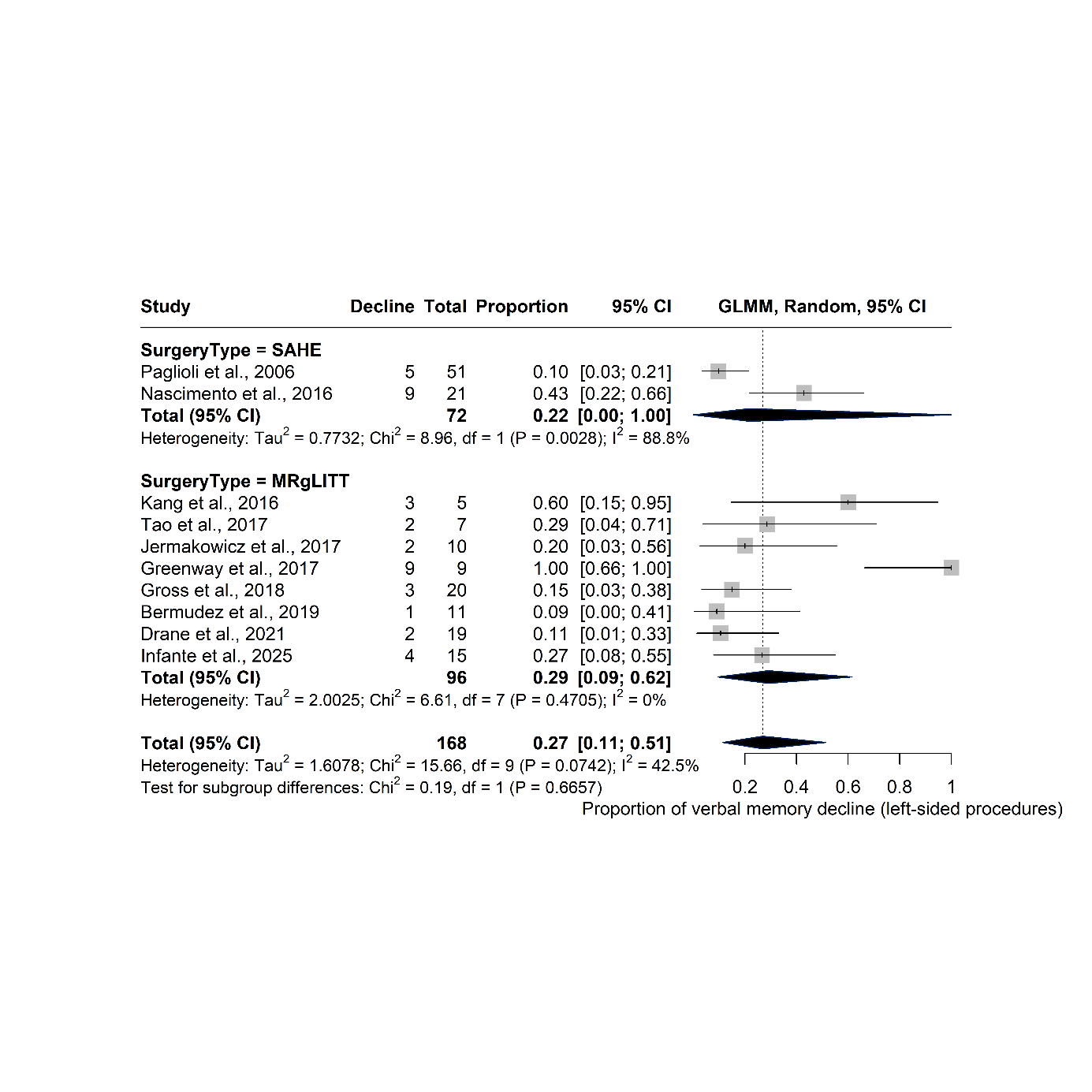
***

Figure S6.4.: Forest plot showing the proportion of verbal memory decline after left-sided procedures, comparing Selective Amygdalohippocampectomy (SAHE) and MRgLITT

S6.5. Subgroup Comparison of ATL and MRgLITT


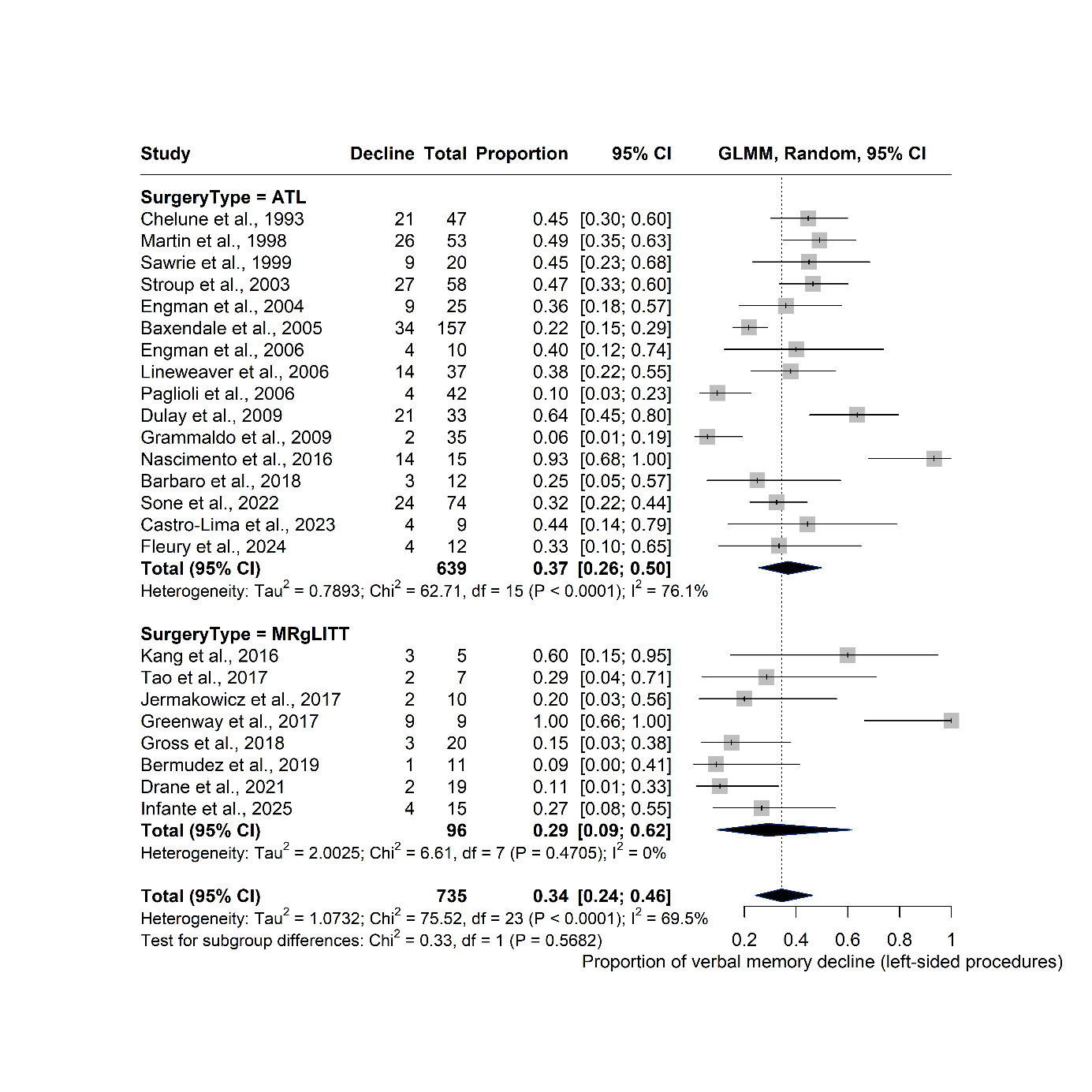


Figure S6.5.: Forest plot showing the proportion of verbal memory decline after left-sided procedures, comparing ATL and MRgLITT.


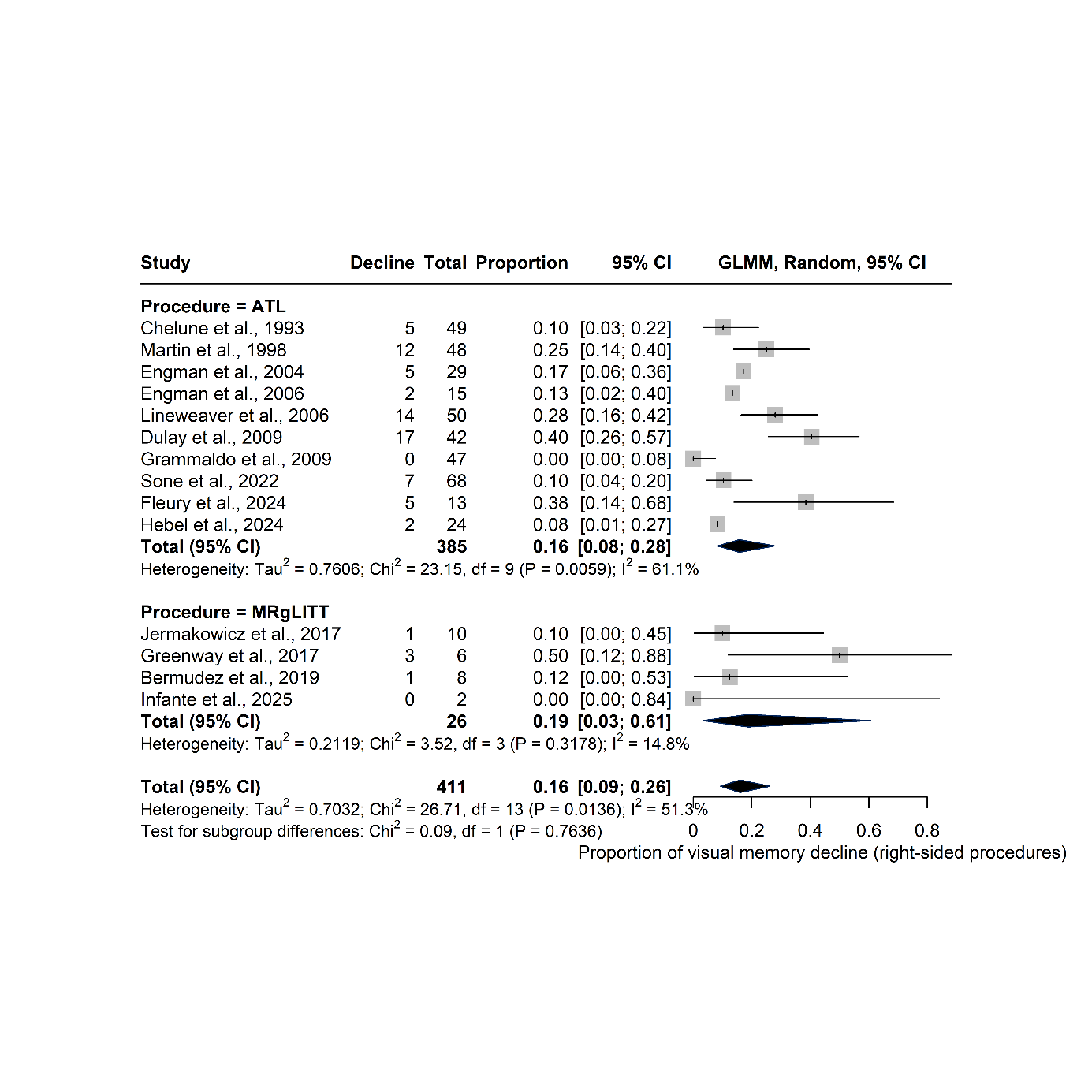


Figure S6.6.: Forest plot showing the proportion of visual memory decline after right-sided procedures, comparing ATL and MRgLITT


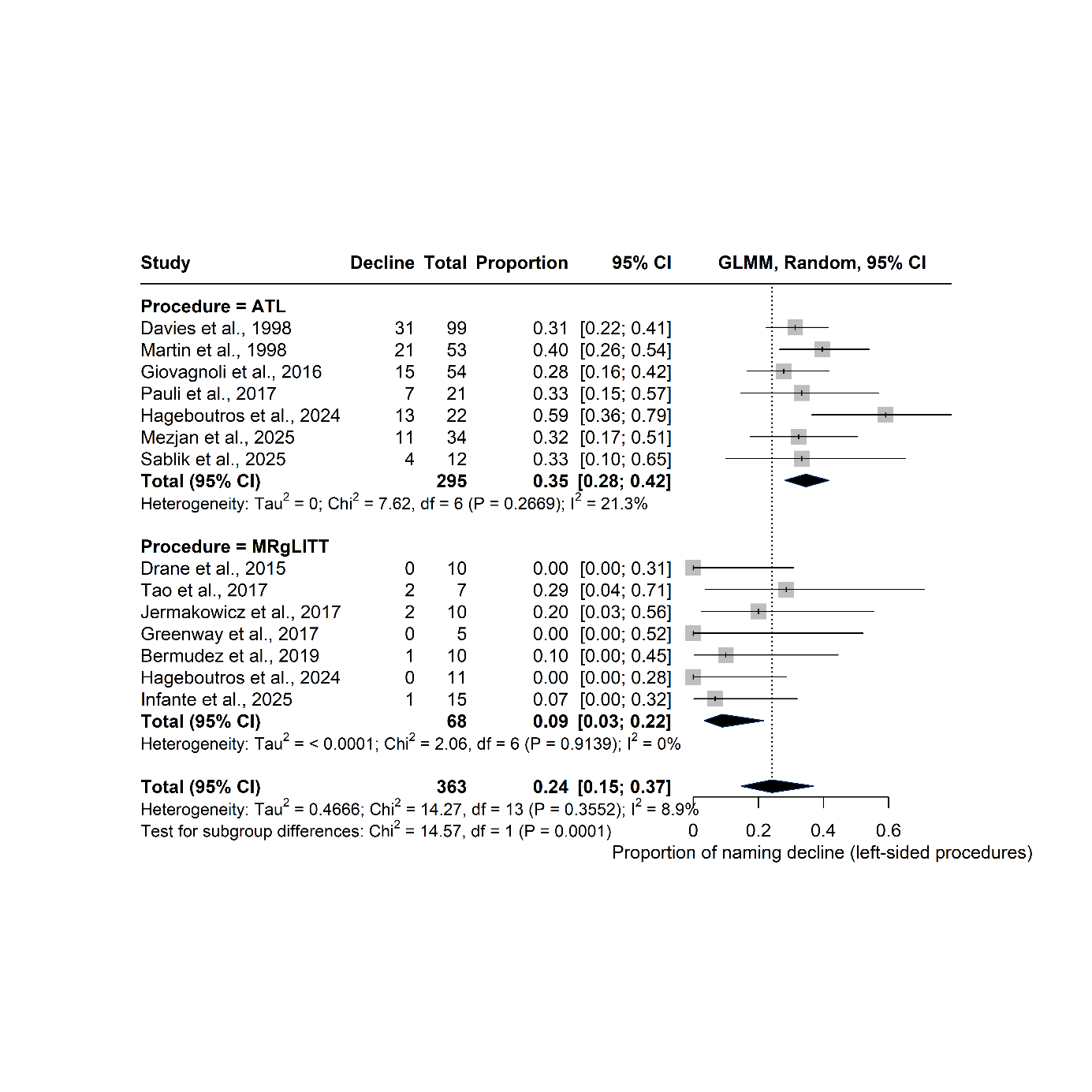


Figure S6.7.: Forest plot showing the proportion of naming decline after left-sided procedures, comparing ATL and MRgLITT
